# Synthesis, Structural Characterization, and Magnetic Behavior of Fe Core–Shell-like Nanoparticles Dispersed in Carbon Matrices

**DOI:** 10.3390/nano16171078

**Published:** 2026-08-29

**Authors:** Vicente Pena Perez, Franco Iglesias, Anand Prakash, Erick Villegas, Armond Khodagulyan, Oscar O. Bernal, Armen N. Kocharian

**Affiliations:** Department of Physics and Astronomy, California State University, Los Angeles, CA 90032, USA; vpenaper@asu.edu (V.P.P.); franco92392@hotmail.com (F.I.);

**Keywords:** macrocyclic precursors, vacuum-sealed thermal conversion, carbon-matrix nanocomposites, dispersed Fe-containing nanostructures, core–shell-like nanoparticles, magnetometry, PXRD, electron microscopy, EDS, skeletonized image connectivity

## Abstract

Metallic and organometallic nanoparticles exhibit intriguing size- and morphology-dependent magnetic properties that differ markedly from their bulk counterparts. Here, we report a detailed synthesis and characterization of iron (Fe), copper (Cu), nickel (Ni), and cobalt (Co) nanostructures dispersed in carbon matrices derived from phthalocyanine (Pc), tetrakis(4-carboxyphenyl)porphyrin (TCPP), and tetraphenylporphyrin (TPP), with the detailed quantitative analysis focused primarily on iron phthalocyanine (FePc), iron tetrakis(4-carboxyphenyl)porphyrin (FeTCPP), and iron tetraphenylporphyrin (FeTPP). Using powder X-ray diffraction (PXRD), scanning electron microscopy (SEM), high-resolution transmission electron microscopy (HRTEM), scanning transmission electron microscopy (STEM), energy-dispersive X-ray spectroscopy (EDS), and magnetometry, we systematically investigate how precursor composition, annealing conditions, and nanostructure formation impact the resulting magnetic behaviors. We introduce a validation-aware image-analysis workflow for morphological, local periodic-contrast, and two-dimensional connectivity descriptors while distinguishing these image-derived quantities from direct measurements of bulk crystallinity, porosity, and three-dimensional connectivity. Hysteresis measurements at low temperatures reveal that iron-based compounds, particularly iron phthalocyanine (FePc) and iron tetrakis(4-carboxyphenyl)porphyrin (FeTCPP), exhibit notable saturation-like magnetization and stronger coercivity, respectively, whereas other precursors (e.g., Cu tetraphenylporphyrin, CuTPP) show weaker magnetic responses. These contrasting behaviors underscore the importance of understanding how candidate phase composition (e.g., metallic Fe, graphite-like carbon, or iron-carbide-related contributions), local morphology, and carbon structure correlate with magnetic characteristics. Microscopy and EDS support Fe-rich regions dispersed within carbonaceous matrices, and a representative Fe/O/C STEM–EDS field provides local evidence for a core–shell-like morphology without establishing a uniform shell thickness, composition, or local core phase across the full population. Our findings highlight the feasibility of tuning carbon–metal nanocomposites through controlled synthesis and post-annealing, thereby motivating future application-specific evaluations in areas such as magnetic hyperthermia, drug delivery, sensing, and electromagnetic materials. The image-analysis workflow also offers a reproducible framework for future studies seeking to relate nanoparticle morphology and magnetic properties to controlled processing.

## 1. Introduction

Nanotechnology has enabled the development of materials whose structural, electronic, and magnetic properties can differ substantially from those of their bulk counterparts. Magnetic nanoparticles are particularly important because finite size, large surface-to-volume ratio, interface structure, and magnetic anisotropy can alter their saturation magnetization, coercivity, remanence, relaxation dynamics, and response to an applied field [1,2,3,4,5,6]. Depending on particle size, magnetic-domain configuration, anisotropy, temperature, and interparticle interactions, Fe-, Co-, and Ni-containing nanoparticles may display ferromagnetic, ferrimagnetic, blocked, or superparamagnetic-like behavior [7,8,9,10,11,12,13,14]. These properties have motivated their investigation for magnetic sensing, information storage, catalysis, magnetic resonance imaging, drug delivery, and magnetic hyperthermia.

Carbon encapsulation provides an additional means of controlling the properties of a magnetic nanoparticle. A surrounding carbonaceous matrix may limit oxidation, reduce direct particle–particle contact, affect aggregation, and modify magnetic interactions at the metal–carbon interface [8,15,16,17]. However, the carbon host cannot always be treated as a magnetically silent coating. Defects, heteroatoms, edges, residual precursor species, stacking disorder, and trace contaminants may produce diamagnetic, paramagnetic, or hysteretic contributions whose magnitude depends on the precursor and processing history [18,19,20,21,22,23,24,25,26,27,28]. Reports of graphite-related ferromagnetism, including the graphite nodule from the Canyon Diablo meteorite, further motivate careful separation of intrinsic and extrinsic magnetic contributions [29]. Consequently, interpreting a metal-containing specimen requires comparison with an experimentally measured metal-free carbon matrix rather than an assumption that the host contribution is zero.

Our previous study established the magnetic response of metal-free phthalocyanine (Pc), tetraphenylporphyrin (TPP), and tetrakis(4-carboxyphenyl)porphyrin (TCPP) matrices produced using a short 10 min thermal-conversion protocol and selected O_2_, N_2_, and Ar post-anneals [30]. Those measurements showed that the matrix response was non-zero and depended on precursor, measurement temperature, and post-annealing atmosphere. In the present work, those data are used as a quantitative host–response gauge. The complete prior matrix tables, the magnetic decomposition framework, and the associated anisotropy and relaxation relations are provided in Appendix A. The former matrix study establishes the scale and variability of the carbon-host contribution, whereas the present work examines the substantially larger and more complex response obtained after incorporating transition-metal centers and extending the thermal-conversion time.

Pc-, TCPP-, and TPP-family macrocycles provide related but chemically distinct molecular frameworks for generating carbon matrices and for positioning transition-metal centers before thermal conversion. Their ligand structure, substituent chemistry, metal coordination, polymorphism, and C/H/N/O composition can influence decomposition pathways, carbon ordering, metal redistribution, and the formation of metallic, carbide, or oxide contributions [31,32,33,34,35]. Magnetic behavior observed in the original molecular precursor cannot be transferred directly to the pyrolyzed material because of high-temperature conversion, which substantially reorganizes both the coordination environment and the carbon framework. Nevertheless, precursor chemistry provides an important processing variable through which the final metal-containing and carbonaceous phases may be influenced.

Recent studies further show that precursor architecture, carbonization conditions, morphology, and metal–carbon interfaces can strongly influence the functional response of magnetic carbon nanocomposites. Relevant examples include Co@N-doped-carbon/Ni, polyphthalocyanine-derived CoFe-alloy/C, carbon-nanotube-decorated FeNi/N-doped carbon, Fe_3_C-containing hierarchical composites, and BiCo@N-doped-carbon architectures [36,37,38,39,40]. These reports focus mainly on electromagnetic-wave attenuation rather than the magnetometry questions examined here, but they provide timely context for the importance of precursor selection, carbon structure, and thermal processing.

Establishing relationships among synthesis, structure, morphology, and magnetism remains challenging because the available characterization techniques probe different spatial scales. Magnetometry measures the collective response of the entire specimen, while powder X-ray diffraction (PXRD) provides bulk-averaged information about crystalline phases and coherent-domain dimensions. Scanning electron microscopy (SEM), transmission electron microscopy (TEM), high-resolution transmission electron microscopy (HRTEM), scanning transmission electron microscopy (STEM), and energy-dispersive X-ray spectroscopy (EDS) provide local morphological and elemental information. A bulk diffraction assignment cannot, therefore, be transferred automatically to an individual object in a microscopy image, and an image-derived diameter cannot automatically be identified with a PXRD coherent-domain size or a magnetically active volume [30,41].

Quantitative image analysis can improve reproducibility when large collections of microscopy fields must be compared. Thresholding, connected-component analysis, ellipse fitting, local Fourier transforms, ridge enhancement, and skeleton-network extraction provide descriptors of size, shape, orientation, periodic contrast, and connectivity [42,43,44,45,46,47]. These descriptors remain dependent on image calibration, segmentation parameters, field selection, and validation rules. In the present study, they are, therefore, reported as image-derived morphological or connectivity descriptors rather than as automatic identification of individual nanoparticles, atomic layers, crystal phases, physical junctions, or three-dimensional porosity [48].

In this work, we investigate carbon-based nanostructures derived from Fe-, Co-, Ni-, and Cu-containing macrocyclic precursors using vacuum-sealed thermal conversion at 900 °C, selected post-annealing treatments, magnetometry, PXRD, SEM, TEM/STEM, EDS, and validation-aware computational image analysis. The detailed quantitative discussion is centered on systems derived from iron phthalocyanine (FePc), iron tetrakis(4-carboxyphenyl)porphyrin (FeTCPP), and iron tetraphenylporphyrin (FeTPP), while the cobalt-, nickel-, and copper-containing TPP-family specimens, labeled CoTPP, NiTPP, and CuTPP, respectively, provide broader comparative context.

The novelty of the present approach is the integration of several levels of evidence within one precursor-to-property framework: the previously measured metal-free Pc/TPP/TCPP matrices are used as an experimental host–response gauge; Fe-containing derivatives from chemically distinct macrocycles are compared under controlled thermal histories; bulk PXRD is interpreted alongside local STEM/EDS without transferring bulk phase labels directly to individual particles; and image-derived morphology/connectivity descriptors are reported with explicit validation and sampling limits. This combined strategy is intended to distinguish precursor, carbon-matrix, phase, and local-morphology contributions more carefully than any one characterization technique can do alone.

This controlled precursor–processing–structure–magnetism framework also provides a natural baseline for future studies of compositionally complex and high-entropy magnetic nanomaterials. Recent work has shown that magnetic high-entropy nanoalloys can exploit multielement chemical disorder, nanoscale interfaces, and controlled precipitation to tune magnetization, permeability, coercivity, and related multifunctional properties [49,50]. A particularly relevant recent example used precursor transformation to generate uniformly dispersed high-entropy alloy nanoparticles anchored to a high-entropy oxide support, demonstrating how precursor chemistry can serve simultaneously as a structural scaffold and compositional reservoir [51]. The materials reported here are not high-entropy phases; rather, the present FePc-, FeTCPP-, and FeTPP-derived systems establish lower-compositional-complexity reference behavior that can guide future mixed-metal precursor or precursor-mixture studies. Any future claim of a high-entropy magnetic phase would require quantitative multielement composition and spatially resolved structural evidence in addition to precursor stoichiometry.

The principal objectives of this study are to (i) use the previously established Pc-, TPP-, and TCPP-derived metal-free matrices as a quantitative gauge for interpreting the magnetic response of the metal-containing materials; (ii) compare the magnetic behavior of FePc-, FeTCPP-, and FeTPP-family-derived materials as functions of measurement temperature, thermal-conversion time, and selected post-annealing conditions; (iii) compare bulk PXRD phase and carbon-ordering descriptors with local microscopy and EDS observations without assigning a bulk phase directly to an individual imaged domain; (iv) evaluate the effects of precursor identity and processing history across the broader Fe-, Co-, Ni-, and Cu-containing specimen set; and (v) implement reproducible morphology and skeleton-network workflows whose outputs are accompanied by explicit validation procedures and clearly defined interpretive limits.

This combined framework permits the metal-free carbon response, the metal-containing magnetic response, and the image-derived structural descriptors to be compared without assuming that any single measurement uniquely determines the magnetic phase, particle architecture, or physical mechanism. Its principal methodological novelty is, therefore, not a single isolated measurement, but the cross-scale, validation-aware comparison of host magnetism, dispersed metal-containing nanostructures, bulk phase information, local elemental morphology, and magnetic response within the same precursor-processing framework.

## 2. Experimental Methods and Techniques

### 2.1. Materials and Nomenclature

The metal-free precursor families were Pc, TCPP, and TPP. Metal-containing derivatives included FePc, FeTCPP, FeTPP, CoTPP, NiTPP, and CuTPP, where the short TPP-family labels preserve historical sample nomenclature and do not imply identical ligand substitution for every metal complex.

### 2.2. Vacuum-Sealed Thermal Conversion and Post-Annealing

Each precursor was loaded into a quartz tube, evacuated to approximately $10^{-4}$ Torr, and sealed with an oxygen–acetylene torch at a position more than 20 cm from the sample. The sealed tube remained closed during conversion at 900 °C for the specified dwell time. Because macrocycle decomposition produces multiple volatile fragments, the reactions are represented only by non-stoichiometric process equations. For a metal-free ligand $H_{2}L$,

(1) $$H_{2}L\left(s\right)\overset{900\;{}^{\circ}C,\;t}{\underset{∼10^{-4}\;\mathrm{Torr}}{\to }}C_{\mathrm{matrix}}\left(s\right)+\sum_{j}\nu_{j}V_{j}\left(g\right),$$

and for a metal complex ML,

(2) $$\mathrm{ML}\left(s\right)\overset{900\;{}^{\circ}C,\;t}{\underset{∼10^{-4}\;\mathrm{Torr}}{\to }}M/M_{x}C_{y}\;\;-\mathrm{containing}\;\mathrm{domains}@C_{\mathrm{matrix}}\left(s\right)+\sum_{j}\nu_{j}V_{j}\left(g\right).$$

Here, $V_{j}$ denotes volatile C/H/N/O-containing fragments; Equations (1) and (2) are schematic and are not balanced chemical equations or universal phase assignments.

Post-annealing was a separate treatment performed on selected samples in flowing O_2_, N_2_, or Ar. The standard treatment used a 3 °C min^−1^ ramp to 150 °C, a 180 min hold, and furnace cooling. Oxidative transformation may be represented generically as

(3) $$M@C+\frac{x}{2}O_{2}\to {\mathrm{MO}}_{x}@C,$$

while N_2_ and Ar are treated as processing atmospheres rather than assigned stoichiometric reactants. Historical PXRD specimens processed at other temperatures and times are labeled directly in the corresponding figure.

### 2.3. Magnetometry

Magnetization was measured using the vibrating sample magnetometer (VSM) option of a Physical Property Measurement System (PPMS). The displayed M(H) loops extend to ±20 kOe and are normalized by total sample mass. The sample was demagnetized and centered before measurement. Values of $M_{s}$, $M_{r}$, $H_{c}$, $M_{r}/M_{s}$, and loop area were extracted with a common Python 3 workflow; archived analysis figures were generated with Matplotlib 3.11.0. Zero-field-cooled (ZFC) and field-cooled (FC) temperature scans, together with field-dependent temperature scans, were collected from approximately 5 to 300 K; the exact sequence file remains the controlling protocol record.

No independent post-conversion measurement of the Fe mass fraction (for example, by inductively coupled plasma optical emission spectroscopy (ICP-OES) or X-ray fluorescence (XRF)) was available for every specimen. Because precursor composition, carbon yield, volatile loss, and oxidation can alter the recovered Fe fraction, comparisons across precursor families are, therefore, total-specimen-mass-normalized composite comparisons and are not interpreted as intrinsic magnetization per gram of Fe. Unless otherwise stated, each processing condition represents one archived synthesis specimen; repeated field points within a loop are not independent synthesis replicates. The numerical descriptors thus characterize the measured record and common extraction procedure rather than sample-to-sample standard deviations.

### 2.4. Powder X-Ray Diffraction and Structural Equations

PXRD data were collected using Bruker D2 PHASER and Panalytical Empyrean diffractometers with Cu Kα radiation; the instrument associated with each dataset is retained in the acquisition record. Interplanar spacing was calculated from Bragg’s law,

(4) $$2d_{hkl}\sin\theta =n\lambda ,$$

and the carbon interlayer spacing is

(5) $$d_{002}=\frac{\lambda }{2\sin\theta_{002}}.$$

The coherent-domain size was estimated using the Scherrer Equation [52,53],

(6) $$L=\frac{K\lambda }{\beta \cos\theta },$$

where β is the full width at half maximum (FWHM) in radians after any stated instrumental correction. For graphitic carbon, the commonly used reflection-specific forms are

(7) $$L_{c}=\frac{K_{c}\lambda }{\beta_{002}\cos\theta_{002}},\;L_{a}=\frac{K_{a}\lambda }{\beta_{100/101}\cos\theta_{100/101}},$$

with the constants reported by the adopted analysis protocol. The numerical notebooks used K=0.9 for the traceable $L_{c}$ values reported here. Crystallinity was calculated from fitted peak areas as

(8) $$X_{c}=\frac{\sum A_{\mathrm{merged}\;\mathrm{crystalline}\;\mathrm{peaks}}}{A_{\mathrm{total}}}\times 100\%,$$

and an analysis-specific graphite-peak intensity fraction was calculated as

(9) $$G_{I}=\frac{\sum I_{\mathrm{graphite}\;\mathrm{peaks}}}{\sum I_{\mathrm{all}\;\mathrm{fitted}\;\mathrm{peaks}}}\times 100\%.$$

Equations (8) and (9) are sensitive to background and peak-merging choices and are, therefore, used only where the fit provenance is reproducible.

Candidate phase assignments were compared with International Centre for Diffraction Data (ICDD) reference patterns for graphite-2H (00-041-1487), graphite-3R (00-026-1079), α-Fe (00-006-0696), γ-Fe (04-002-3692), Fe_3_C (00-035-0772), Fe_3_O_4_ (00-019-0629), and α-Fe_2_O_3_ (00-033-0664). Because several reflections overlap or broaden in carbon-rich nanocomposites, these references guide indexing but do not establish unique phase identification or phase fractions where peaks cannot be resolved.

### 2.5. Electron Microscopy, EDS, and Image Analysis

Selected scanning electron microscopy (SEM), transmission electron microscopy (TEM), scanning transmission electron microscopy (STEM)/high-angle annular dark-field (HAADF), and energy-dispersive X-ray spectroscopy (EDS) images were used to characterize morphology and elemental contrast. A high-contrast center surrounded by carbonaceous contrast is described operationally as a core–shell or core–shell-like morphology; this does not by itself establish the local core phase, oxidation state, shell stoichiometry, or uniform shell thickness. A representative high-magnification Fe/O/C STEM–EDS overlay is included in the Results to provide direct local evidence for elemental differentiation in one core–shell-like object. Because the rendered EDS channels are qualitative rather than common-scale concentration maps, this local example is not used to claim a uniform architecture, quantitative shell composition, or population-average shell thickness for all particles.

For the skeletonized image-connectivity workflow, each calibrated source field was normalized, fast Fourier transform (FFT) bandpass filtered, background corrected, denoised, enhanced with contrast-limited adaptive histogram equalization (CLAHE) and a Sato ridge filter, thresholded, morphologically cleaned, thinned, and spur-pruned. The field was, therefore, skeletonized first using a fixed parameter set; sensitivity checks were then used to validate the resulting descriptors rather than altering the image selectively during construction. This validation-aware implementation was informed by established quantitative HRTEM morphology and modern statistical electron-microscopy image-analysis approaches [42,43,54,55,56,57]. Adjacent branch pixels were clustered into graph nodes, and ordered residual paths were retained as graph edges. The resulting graph describes two-dimensional line-like image contrast and is not treated as a direct measurement of crystallinity, bulk porosity, physical junctions, or three-dimensional connectivity. For edge *e*,

(10) $$\begin{array}{cc} L_{e} & =\sum_{i=1}^{N_{e}-1}∥r_{i+1}-r_{i}∥, \end{array}$$

(11) $$\begin{array}{cc} C_{e} & =∥r_{N_{e}}-r_{1}∥, \end{array}$$

(12) $$\begin{array}{cc} \tau_{e} & =\frac{L_{e}}{C_{e}}\geq 1,\;S_{e}=\frac{C_{e}}{L_{e}}=\tau_{e}^{-1}\leq 1. \end{array}$$

For field-balanced histograms, each edge in field *i* received weight

(13) $$w_{ij}=\frac{1}{N_{\mathrm{fields}}n_{i}}.$$

The validated FeTCPP cohort contained three non-post-annealed fields, eight O_2_-post-annealed fields, and one N_2_-post-annealed field. Microscopy fields, rather than individual graph edges, are the independent imaging units. The N_2_ condition is, therefore, retained for descriptive comparison only and is not used to support a strong treatment-level statistical conclusion.

## 3. Materials and Synthesis

### 3.1. Precursor Chemistry

Pc, TCPP, and TPP-family molecules differ in ring fusion, peripheral substituents, heteroatom content, and metal coordination. These differences alter the volatile inventory released during vacuum-sealed thermal conversion and influence carbon yield, local ordering, defect chemistry, and metal redistribution. The molecular structures define the starting materials; they are not assumed to survive intact after heating to 900 °C.

### 3.2. Processing Matrix

The metal-free 10 min series is retained from the previous article as the introductory host–response gauge [30]; it is not pooled with the present Fe-containing series. The current nanoparticle comparison emphasizes 60, 120, and 180 min conversion, with selected O_2_, N_2_, or Ar post-anneals. The 60 min condition was retained as the principal comparative condition because it produced strong magnetic responses in FePc and FeTCPP while limiting the additional structural evolution observed at longer dwell times.

## 4. Results and Discussion

### 4.1. Magnetic Properties of Dispersed Fe-Containing Core–Shell-like Nanostructures

#### 4.1.1. FePc

FePc exhibits the largest verified mass-normalized magnetization in the Fe series. At 10 K, the 60, 120, and 180 min samples show $M_{s}$ values of approximately 16–21 emu g^−1^ and coercivities of approximately 0.69–1.15 kOe. At 300 K, $M_{s}$ remains 15.9–19.0 emu g^−1^ while $H_{c}$ decreases to approximately 0.29–0.39 kOe. N_2_ post-annealing changes the loop parameters but does not erase the hysteretic response.

#### 4.1.2. FeTCPP

FeTCPP shows a lower saturation-like magnetization than FePc but a broader low-field loop. The 60 min non-annealed, N_2_-annealed, and O_2_-annealed samples all have $M_{r}/M_{s}\approx 0.38$ at 10 K, with coercivities near 1.9 kOe. The 180 min sample has a larger $M_{s}$ but lower remanence ratio and coercivity, indicating a processing-dependent change in magnetic reversal behavior.

#### 4.1.3. FeTPP

**Figure 1:**
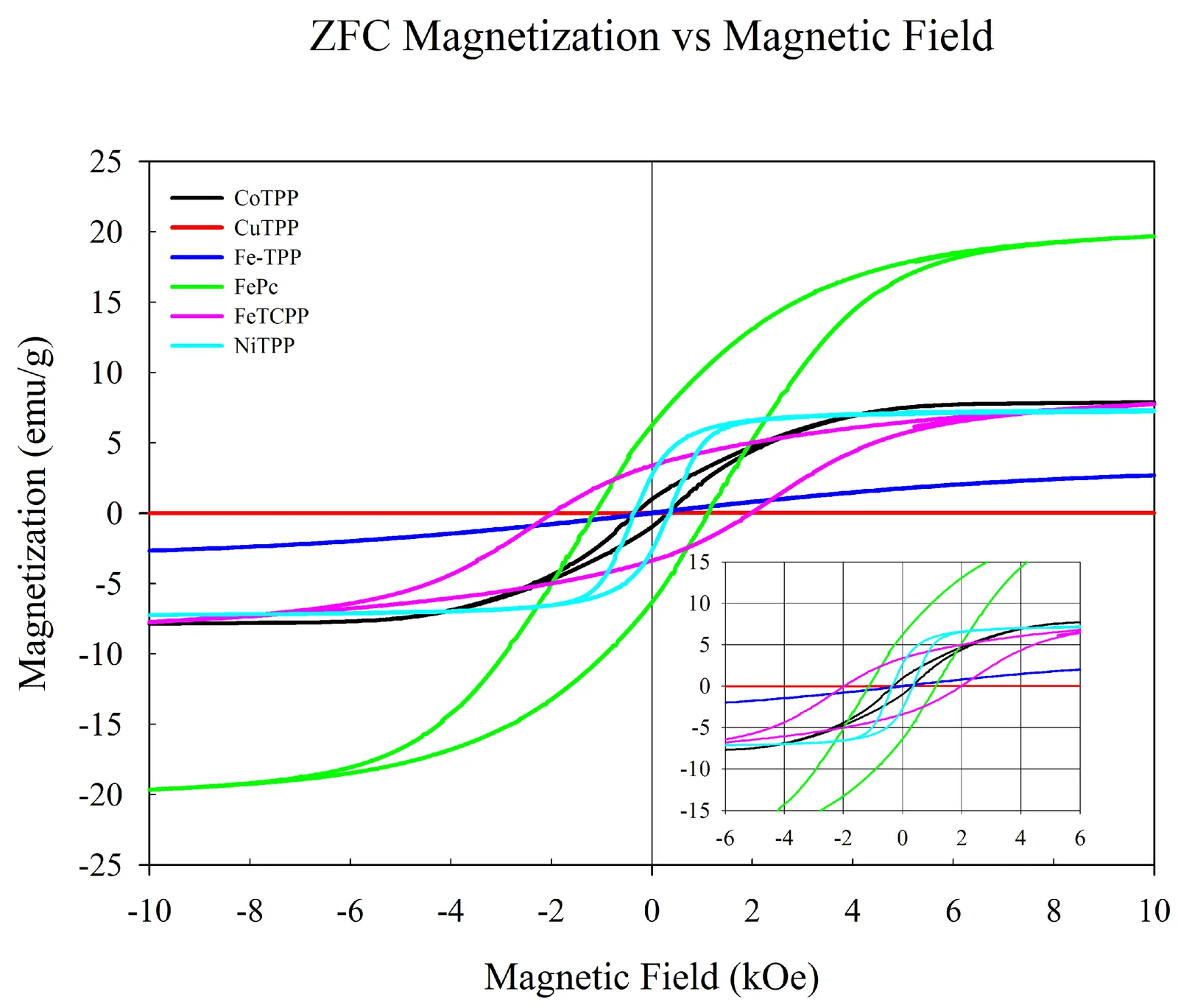
Mass-normalized ZFC magnetic hysteresis loops for the available CoTPP-, CuTPP-, FeTPP-, FePc-, FeTCPP-, and NiTPP-derived specimens. Unless otherwise indicated, samples were thermally converted at 900 °C for 60 min; CoTPP was converted for 120 min. FeTPP, FePc, FeTCPP, and NiTPP were measured at 10 K, whereas CoTPP and CuTPP were measured at 300 K. The inset enlarges the low-field region to show differences in coercive width and remanence. Magnetization is normalized by total specimen mass; because measurement temperature and dwell time are not fully matched, the panel is a qualitative multimetal overview rather than a controlled metal-only ranking.

The available FeTPP 60 min specimen has a small coercivity and remanence compared with FePc and FeTCPP. The 10 K and 300 K loops are, therefore, described as weakly hysteretic or nearly paramagnetic-like under the measured conditions rather than as evidence for a robust ferromagnetic state. Figure 1 provides the broader multimetal comparison that motivates the detailed Fe-centered analysis below. Because the available measurement temperatures and dwell times are not identical for every material, it is used as qualitative context rather than as a controlled ranking of intrinsic metal magnetism.

**Figure 2:**
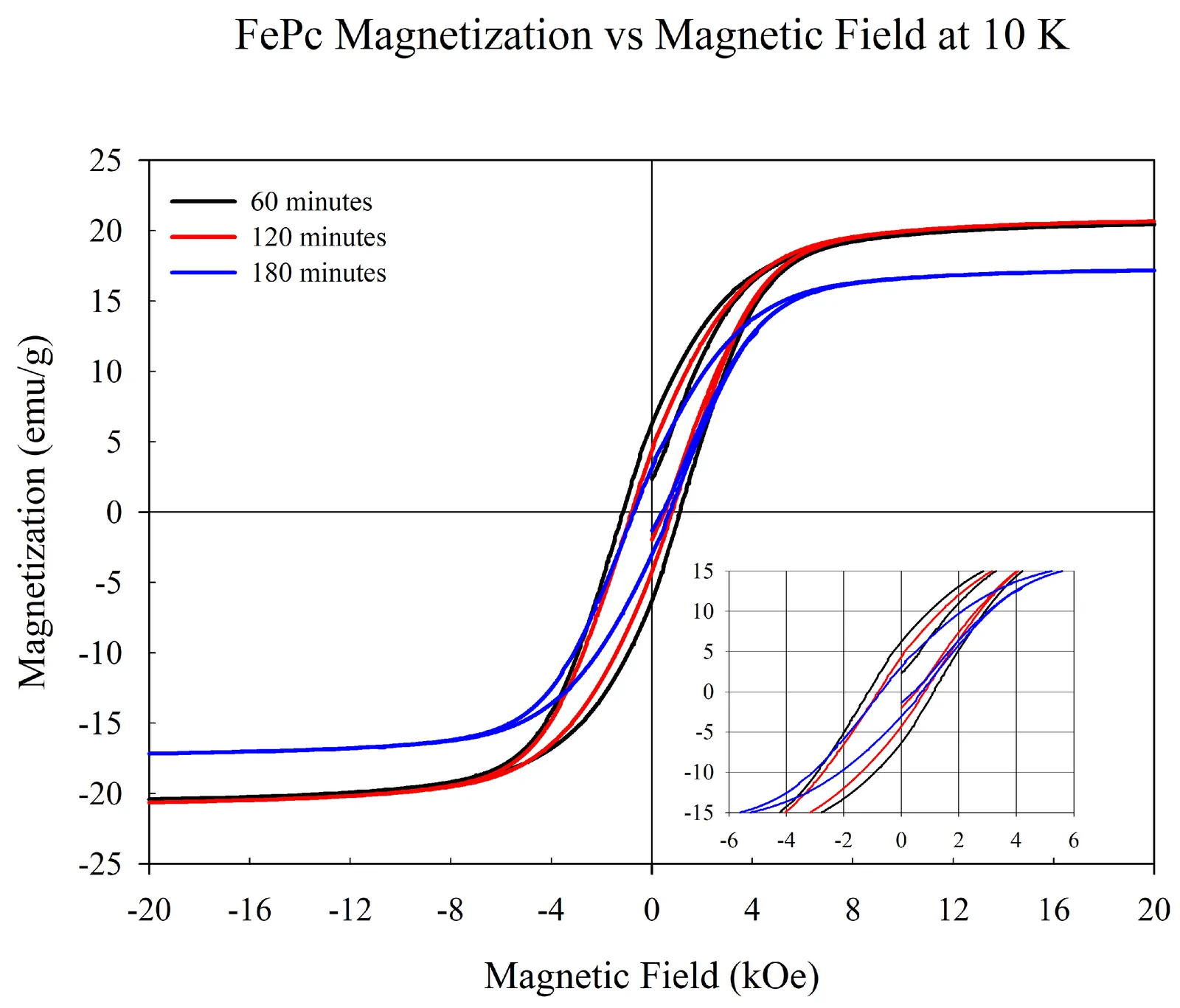
Magnetization versus applied magnetic field for FePc; pyrolysis times are indicated. Measurements were performed at 10 K.

**Figure 3:**
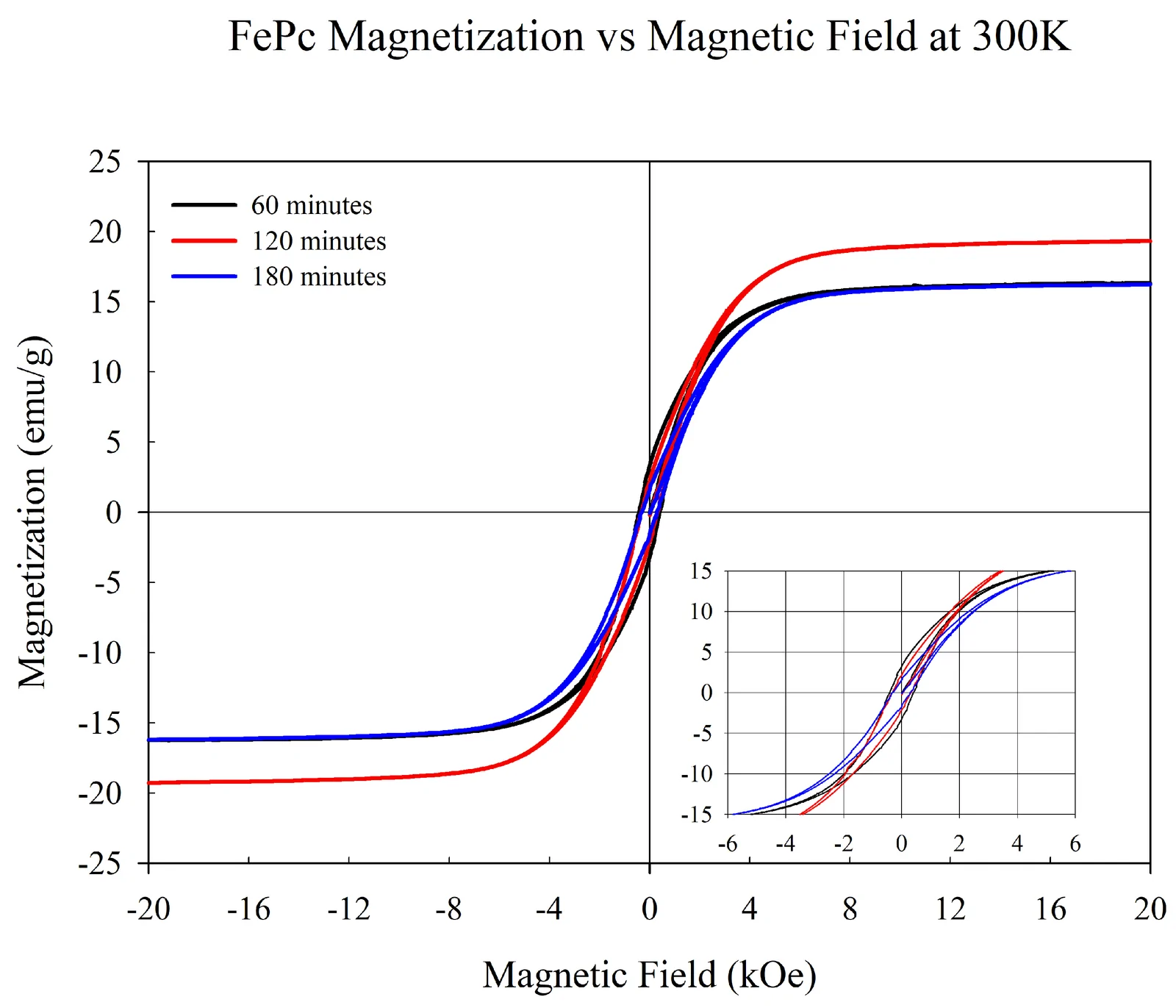
Same as Figure 2, but measurements were performed at 300 K.

**Figure 4:**
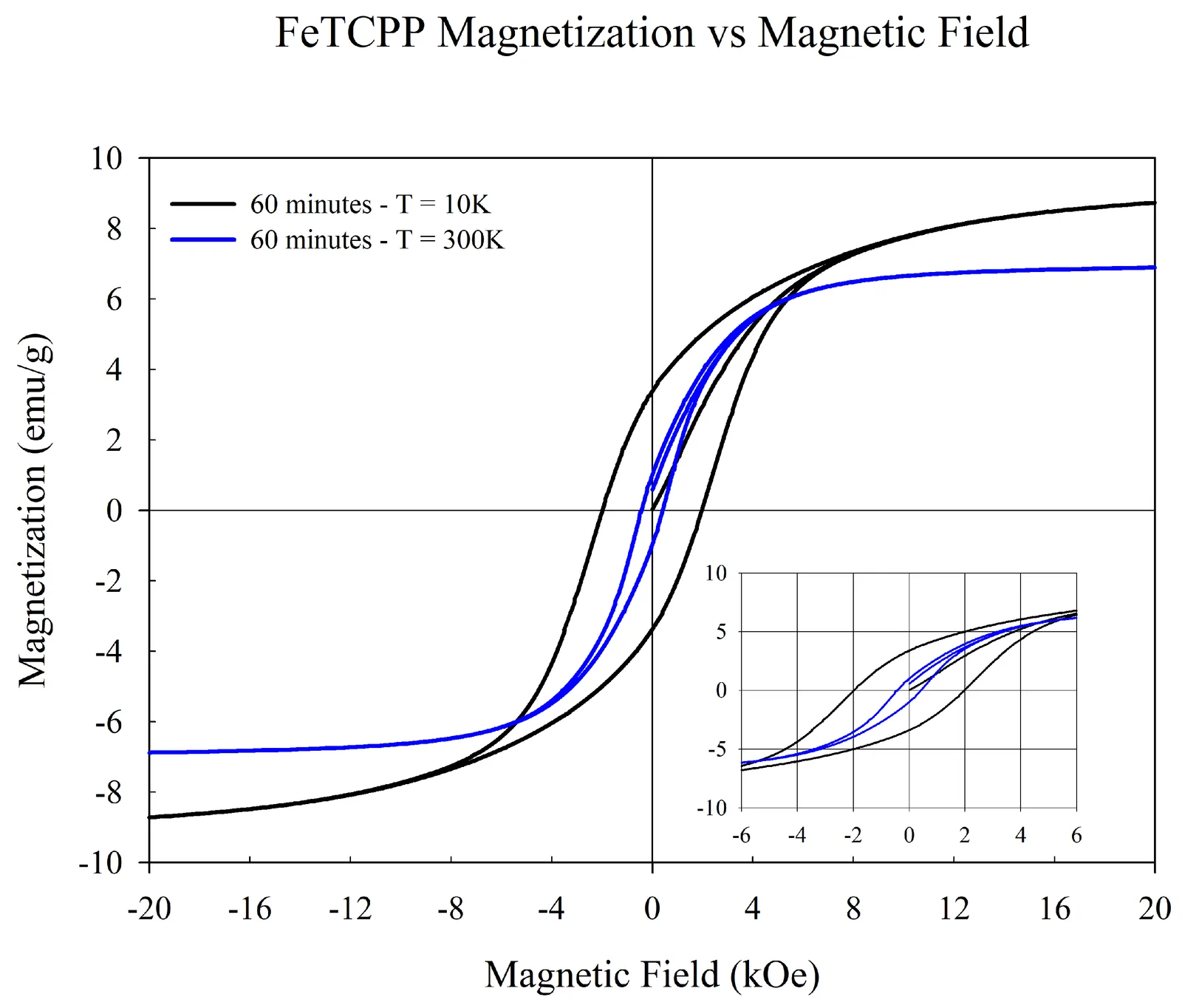
Magnetization versus applied magnetic field for FeTCPP pyrolyzed at 900 °C; measurements were performed at 10 K and 300 K.

**Figure 5:**
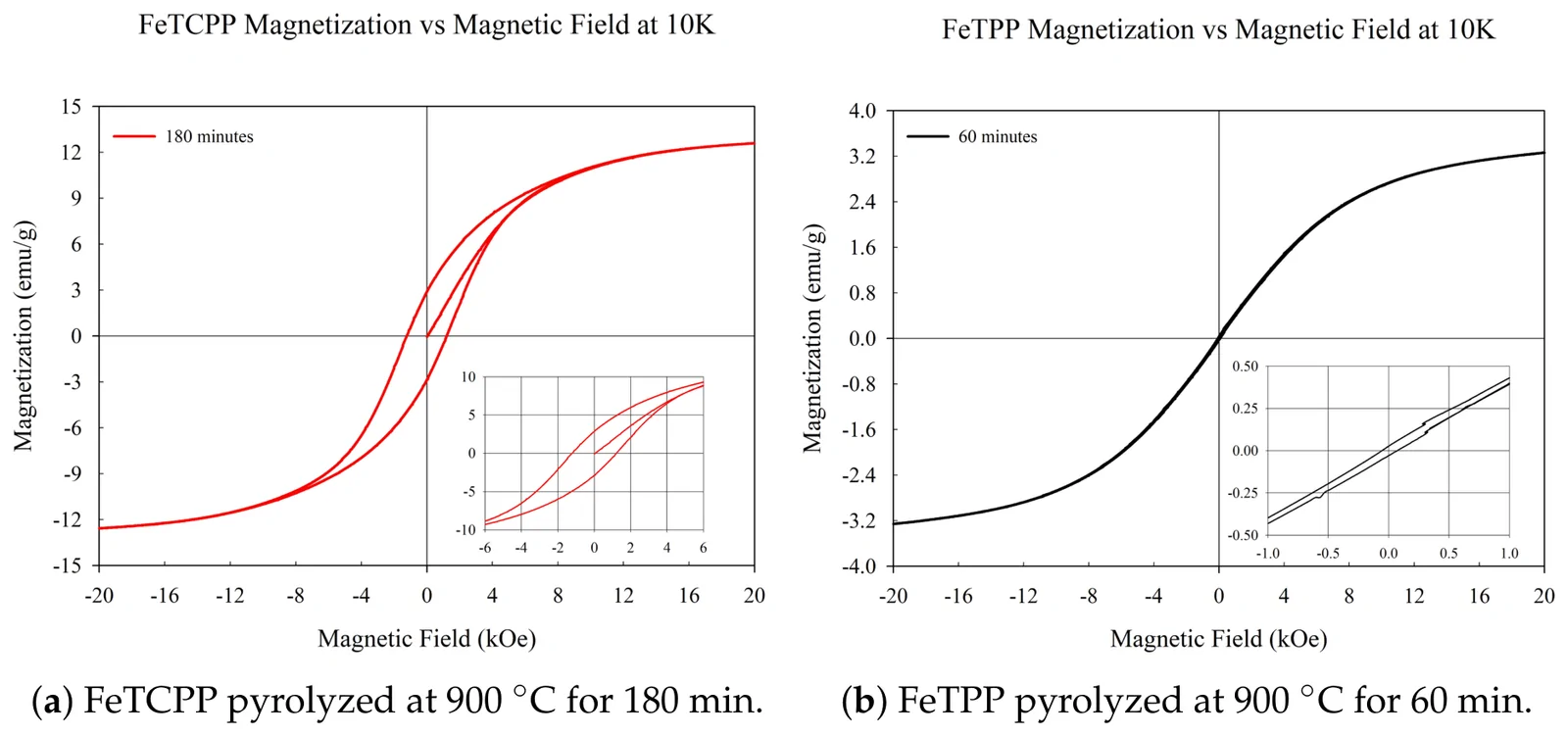
Magnetization versus applied magnetic field; measurements were performed at 10 K.

Figure 2, Figure 3, Figure 4 and Figure 5 separate the FePc and FeTCPP series from the earlier six-panel full-field/low-field composite. The full-range curves show the approach toward saturation-like behavior, while the insets magnify the low-field region used to compare coercive width and remanence. The FePc series compares three conversion times at both 10 and 300 K; the FeTCPP series contains 60 and 180 min measurements.

Figure 2 and Figure 3 show the magnetization versus applied magnetic field for FePc at 10 K and 300 K, respectively. At 10 K, it is observed that the saturation-like magnetization of the 60 min sample closely resembles that of the 120 min sample near 20 emu/g, while the 180 min sample is near 16 emu/g, as reported in Table 1. The coercivities for all samples in Figure 2 range from approximately 0.7 kOe to 1 kOe, as shown by the wider gap near H=0. At 300 K, however, a direct comparison reveals that the saturation-like magnetization of the 60 min sample more closely matches that of the 180 min sample near 16 emu/g, indicating a lower 60 min value at the higher measurement temperature. Additionally, the coercivities for all samples in Figure 3 decrease to a range of 0.28 kOe to 0.39 kOe, narrowing the gap at H=0.

The extracted FePc, FeTCPP, and FeTPP loop descriptors discussed above are summarized in Table 1, Table 2 and Table 3.

Figure 4 shows the magnetization versus magnetic field for FeTCPP at 10 K and 300 K. At 10 K, the 60 min sample displays a magnetic saturation near 8.7 emu/g and a coercivity at approximately 2 kOe. At 300 K, the 60 min sample displays a smaller magnetic saturation near 7 emu/g and a smaller coercivity at approximately 0.5 kOe.

Figure 5 compares two different samples measured at 10 K and prepared under different conditions: FeTCPP pyrolyzed for 180 min (Figure 5a) and FeTPP pyrolyzed for 60 min (Figure 5b). FeTCPP at 180 min shows a larger saturation-like magnetization near 12.5 emu/g and a coercivity of approximately 1.20 kOe. When directly compared with FeTPP at 60 min, the magnetization is smaller at approximately 3.2 emu/g and the coercive width is much narrower. Although FeTCPP and FeTPP have related porphyrin-derived precursor frameworks, FeTCPP contains oxygen-bearing carboxyphenyl substituents whereas FeTPP does not; this compositional difference may influence the pyrolysis products and magnetic reversal behavior, but the present data do not establish a unique phase-specific origin for that difference.

#### 4.1.4. Temperature Dependence and ZFC/FC Irreversibility

**Figure 6:**
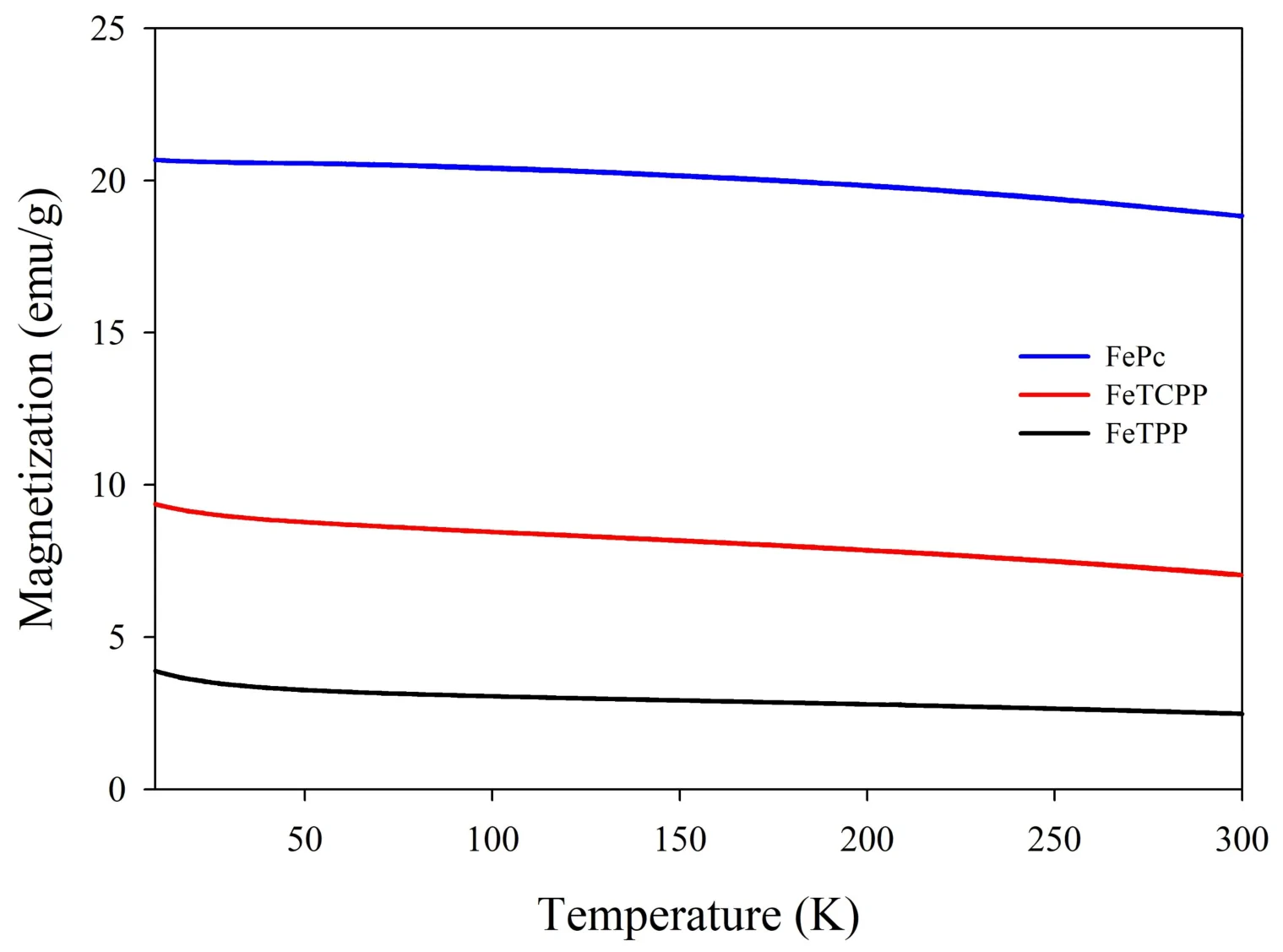
Mass-normalized magnetization as a function of temperature under an applied field of 60 kOe for FePc (blue), FeTCPP (red), and FeTPP (black), over the displayed range from approximately 5 to 300 K. FePc has the largest magnetization throughout the interval, followed by FeTCPP and FeTPP. Each trace decreases smoothly with increasing temperature; the plotted behavior is reported without assigning a unique magnetic phase transition.

**Figure 7:**
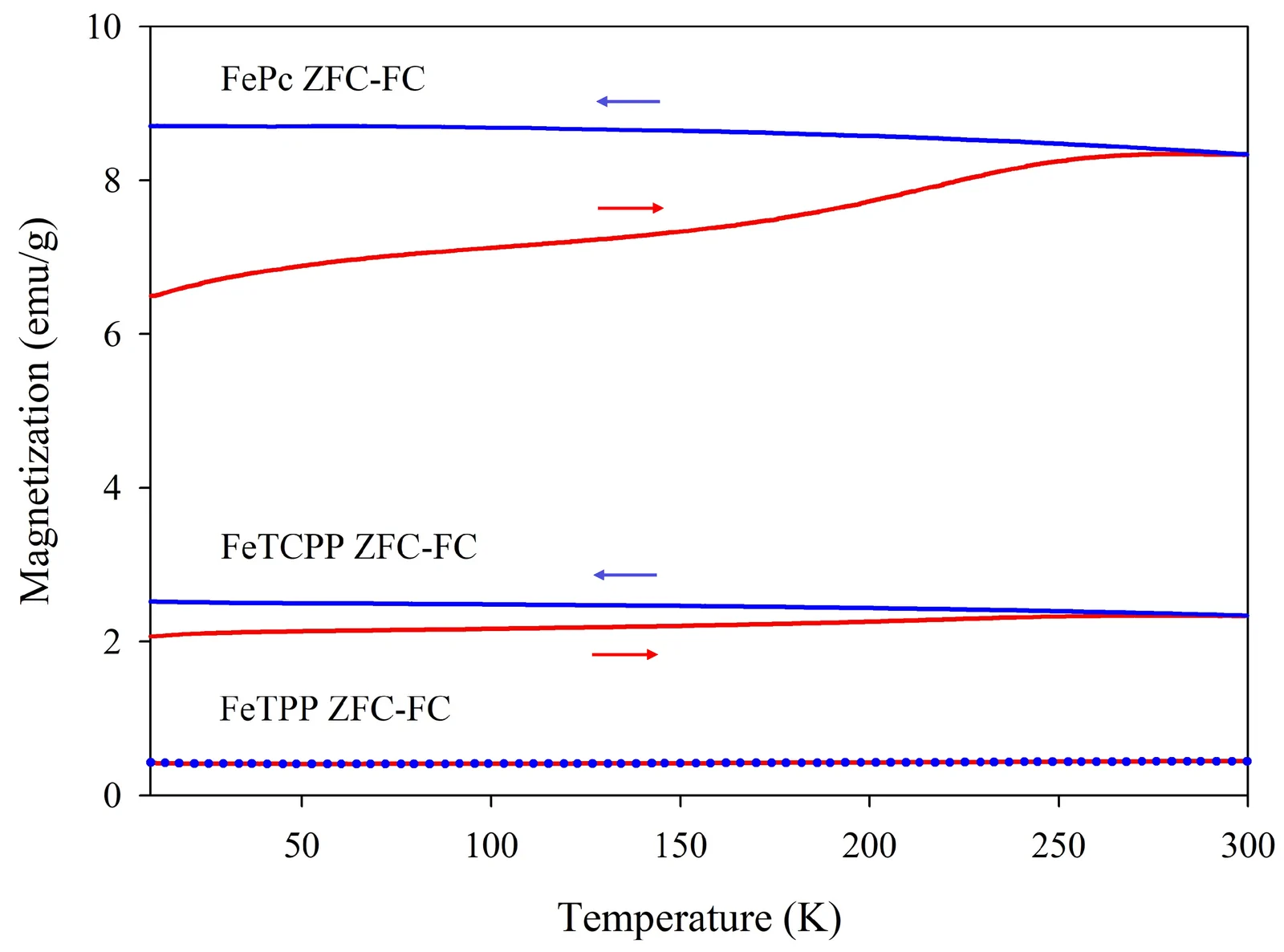
Zero-field-cooled and field-cooled magnetization as a function of temperature at 1 kOe for FePc, FeTCPP, and FeTPP. Blue traces correspond to FC data and red traces to ZFC data; the arrows reproduce the acquisition directions.

The 60 kOe temperature scans decrease smoothly with increasing temperature for FePc, FeTCPP, and FeTPP (Figure 6). The lower-field ZFC/FC curves show the largest separation for FePc, a smaller separation for FeTCPP, and little separation for FeTPP at the plotted scale (Figure 7). These observations are consistent with thermally activated reversal, distributed anisotropy barriers, and interactions; however, a blocking temperature is not extracted because measurements at higher temperatures would be needed.

Figure 6 shows the zero-field-cooled (ZFC) and field-cooled (FC) magnetization, M(T), curves for FePc, FeTCPP, and FeTPP measured at a strong magnetic field of H=60 kOe, where both ZFC and FC regimes fully coincide. Two key physical parameters can be typically extracted from these ZFC-FC measurements: (i) the irreversibility temperature, $T_{\mathrm{irr}}$, defined as temperature above which $M_{\mathrm{ZFC}}\left(T\right)$ and $M_{\mathrm{FC}}\left(T\right)$ coincide, and (ii) the blocking temperature, ($T_{B}$), where the $M_{\mathrm{ZFC}}\left(T\right)$ curve displays a maximum. It is well established that $T_{\mathrm{irr}}$ and $T_{B}$ are approximately identical for an ideal ensemble of non-interacting, monodisperse superparamagnetic nanoparticles.

In the present study, $M_{\mathrm{ZFC}}\left(T\right)$ and $M_{\mathrm{FC}}\left(T\right)$ curves for FePc, FeTCPP, and FeTPP at 60 kOe overlap across the displayed temperature range, indicating little measurable ZFC–FC irreversibility under this strong applied field within the resolution of the plotted data.

In contrast, at a moderate magnetic field (H=1 kOe), as shown in Figure 7, the behavior differs appreciably. FePc exhibits the largest ZFC–FC separation, FeTCPP a smaller separation, and FeTPP nearly overlapping branches at the plotted scale. The separation is described as magnetic irreversibility; no unique blocking temperature or phase transition is assigned directly from the measured curves alone. For FePc and FeTCPP, the high-temperature ZFC and FC branches approach one another near the upper experimental limit. Because the measurements terminate near 300 K before a complete ZFC maximum is observed, Appendix B.1 treats the branch closure as an explicitly model-dependent extrapolation. Under the stated cubic-fit procedure, the extrapolated closure temperatures are 300.60 K for FePc and 304.78 K for FeTCPP; these values are not directly observed ZFC maxima. FeTPP remains nearly coincident in the plotted 1 kOe data and, therefore, does not support an analogous extracted closure in this analysis.

Overall, this comparative study of temperature-dependent magnetization highlights the strong field dependence of magnetic irreversibility in the Fe-containing specimens. At 60 kOe, the ZFC and FC curves overlap across the displayed range, whereas at 1 kOe FePc and FeTCPP show clear branch separation and FeTPP remains nearly coincident at the plotted scale. Because the measurements end near 300 K, the present data do not directly capture a complete ZFC maximum for FePc or FeTCPP; the near-room-temperature closure values discussed in Appendix B.1 are, therefore, extrapolated estimates under the stated fit model. These field-dependent trends provide a useful magnetic comparison among the dispersed Fe-containing nanocomposites without requiring a unique phase-transition assignment from the available temperature window.

### 4.2. Structural Characterization

#### 4.2.1. PXRD Comparison and Phase Context

**Figure 8:**
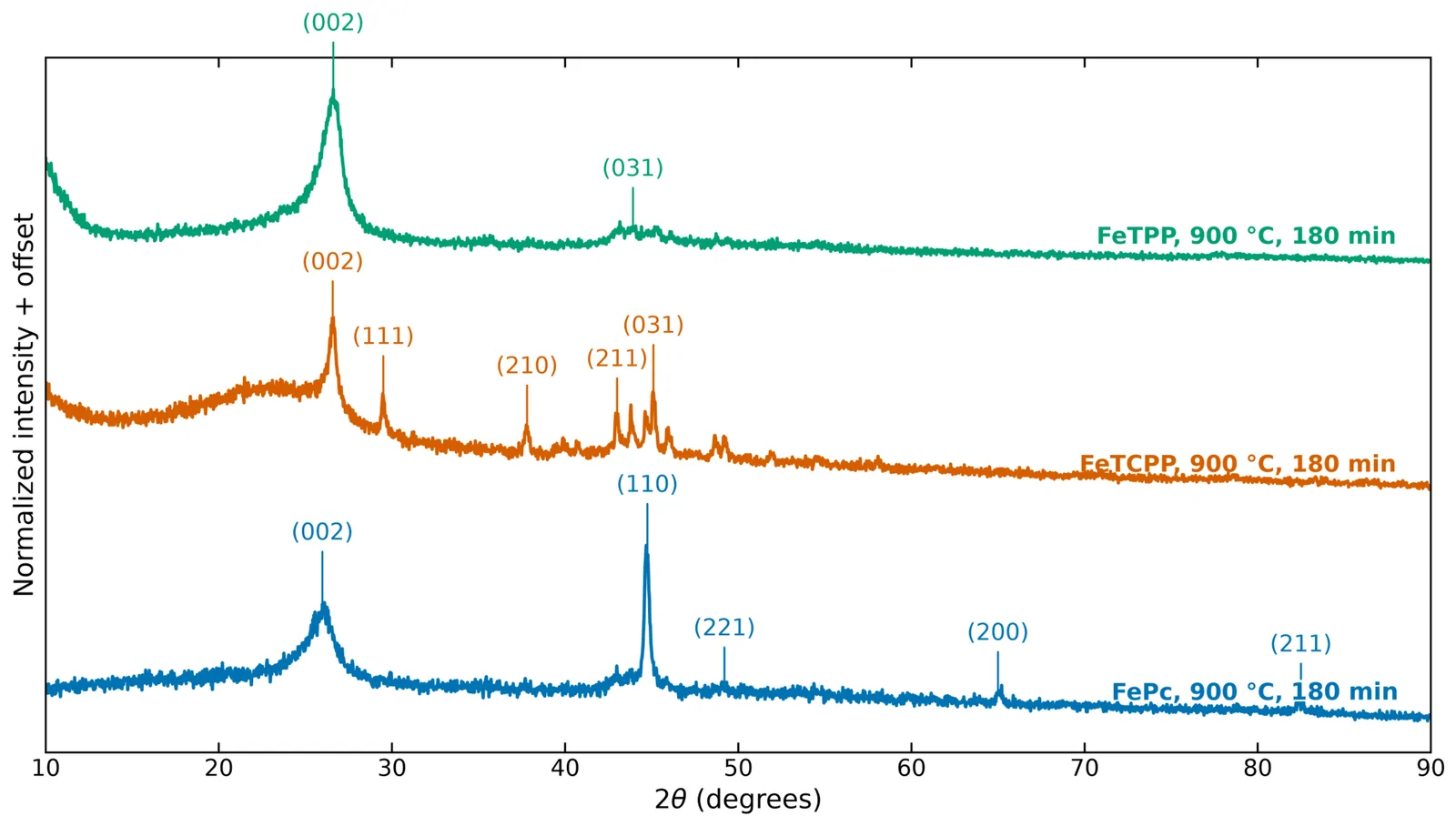
Normalized and vertically offset PXRD patterns of FePc-, FeTCPP-, and FeTPP-derived materials after vacuum-sealed thermal conversion at 900 °C for a 180 min duration. The broad basal feature near $26^{\circ }2\theta$ is assigned to graphite-like carbon. In FePc, the (110), (200), and (211) reflections are assigned to candidate α-Fe, whereas (221) is assigned to candidate cementite, Fe_3_C. In FeTCPP, the (111), (210), (211), and (031) reflections are assigned to candidate Fe_3_C. For FeTPP, the basal carbon reflection is represented by graphite-3R in the phase model, while the feature near 43°–45° may contain overlapping graphite-3R, γ-Fe (111), and α-Fe (110) contributions and is not uniquely phase-resolved.

The 180 min FePc, FeTCPP, and FeTPP patterns contain a broad graphite-like (002) contribution and sharper precursor-dependent reflections (Figure 8). The indexed Fe-containing reflections provide bulk phase context.

Figure 8 shows PXRD data for FePc, FeTCPP, and FeTPP precursors pyrolyzed at 900 °C for 180 min. The broad feature indexed as (002) in each pattern is consistent with a graphite-like carbon contribution and reflects stacking-related order perpendicular to carbonaceous layers. For the FePc-derived pattern, the additional labels (110), (221), (200), and (211) identify the sharper reflections. For the FeTCPP-derived pattern, the labels (111), (210), and (211) identify the corresponding candidate-indexed sharper features. For the FeTPP-derived pattern, (031) denotes an additional candidate-indexed reflection beyond the graphite-like (002) feature. All non-(002) labels are candidate Miller-index assignments used to describe the bulk PXRD patterns.

A cautious interpretation of these patterns is that the FePc-derived specimen contains comparatively stronger and more ordered Fe-related crystalline contributions, whereas the candidate carbide-related contributions in FeTCPP are less strongly ordered and the FeTPP-derived pattern contains weaker or more poorly resolved Fe-related diffraction features. Size ranges reported for similar carbon materials can provide context, but they are not direct particle-size measurements for the present specimens. Accordingly, the PXRD coherent-domain trends are not used here to assign a unique physical nanoparticle diameter or to conclude that a particular imaged domain is amorphous or atomically dispersed.

**Figure 9:**
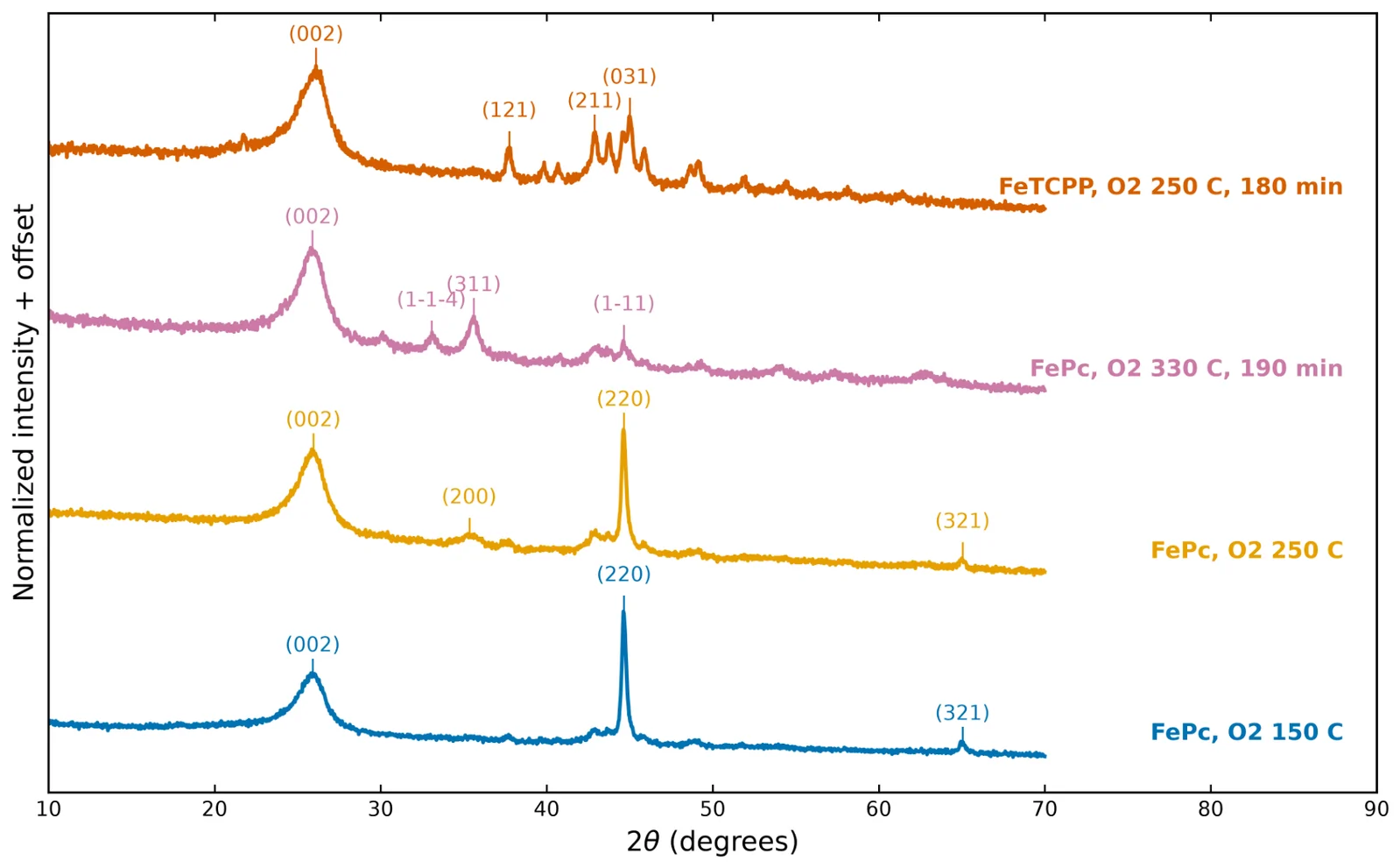
Normalized and vertically offset PXRD patterns of selected O_2_-post-annealed FeTCPP- and FePc-derived materials. The broad feature near $26^{\circ }$2θ is assigned to graphite-like carbon. For FeTCPP post-annealed at 250 °C, the (121), (211), and (031) reflections are assigned to candidate cementite, Fe_3_C. For FePc post-annealed at 330 °C, the labeled reflections are consistent with hematite, α-Fe_2_O_3_, and magnetite, Fe_3_O_4_, including the characteristic magnetite (311) contribution. For FePc post-annealed at 250 °C, the region near $35.5^{\circ }$ contains overlapping candidate Fe_3_C (200) and Fe_3_O_4_ (311) contributions, while the higher-angle features contain overlapping Fe_3_C and α-Fe reflections. The FePc pattern post-annealed at 150 °C contains graphite-like carbon together with candidate Fe_3_C and α-Fe contributions.

Figure 9 shows PXRD data for $O_{2}$ annealed precursors: FeTCPP, O_2_, 250 °C for 180 min; FeTCPP, O_2_, 330 °C for 190 min; FePc, O_2_, 250 °C; and FePc, O_2_, 150 °C. In every pattern, the broad (002) feature is consistent with graphite-like carbon and describes stacking-related carbon order. For FeTCPP annealed at 250 °C, the labeled sharper features are (121), (211), and (031); for FeTCPP annealed at 330 °C, the labels are $\left(1\bar{1}4\right)$, (311), and $\left(1\bar{1}1\right)$. For FePc annealed at 250 °C, the labels are (200), (220), and (321); for FePc annealed at 150 °C, the labels are (220) and (321). This figure shows how precursor identity and post-annealing conditions are associated with changes in the bulk diffraction response.

The post-annealed FePc and FeTCPP patterns are consistent with substantial evolution of the Fe-containing and carbon-related diffraction features as temperature is increased. The FePc pattern at 150 °C retains comparatively sharper Fe-related features, while the 250 °C pattern contains overlapping candidate metallic/carbide/oxide contributions. At the higher post-annealing temperature, broader and flatter features are consistent with increased structural disorder and/or redistribution among Fe-containing phases, but PXRD alone does not establish a specific nanoparticle-fragmentation mechanism, surface-area change, or magnetic state. The FeTCPP patterns likewise show broader candidate carbide/oxide-related contributions in the 2θ≈35–45° region. These differences support precursor- and post-anneal-dependent phase evolution, while the overlapping reflections prevent a unique assignment of oxidation pathway or phase fraction from the present patterns alone.

#### 4.2.2. Carbon Structural Parameters

The PXRD-derived carbon-ordering metrics are summarized in Table 4 and Table 5; these values are coherent-domain and fit-dependent descriptors rather than direct particle-size or bulk-porosity measurements.

The FePc $d_{002}$ values increase slightly with dwell time, while $L_{c}$ rises most strongly in the 180 min record. FeTCPP remains near $d_{002}\approx 0.334$–0.335 nm, but its recovered $L_{c}$ is non-monotonic.

### 4.3. Morphological Characteristics and Elemental Composition

**Figure 10:**
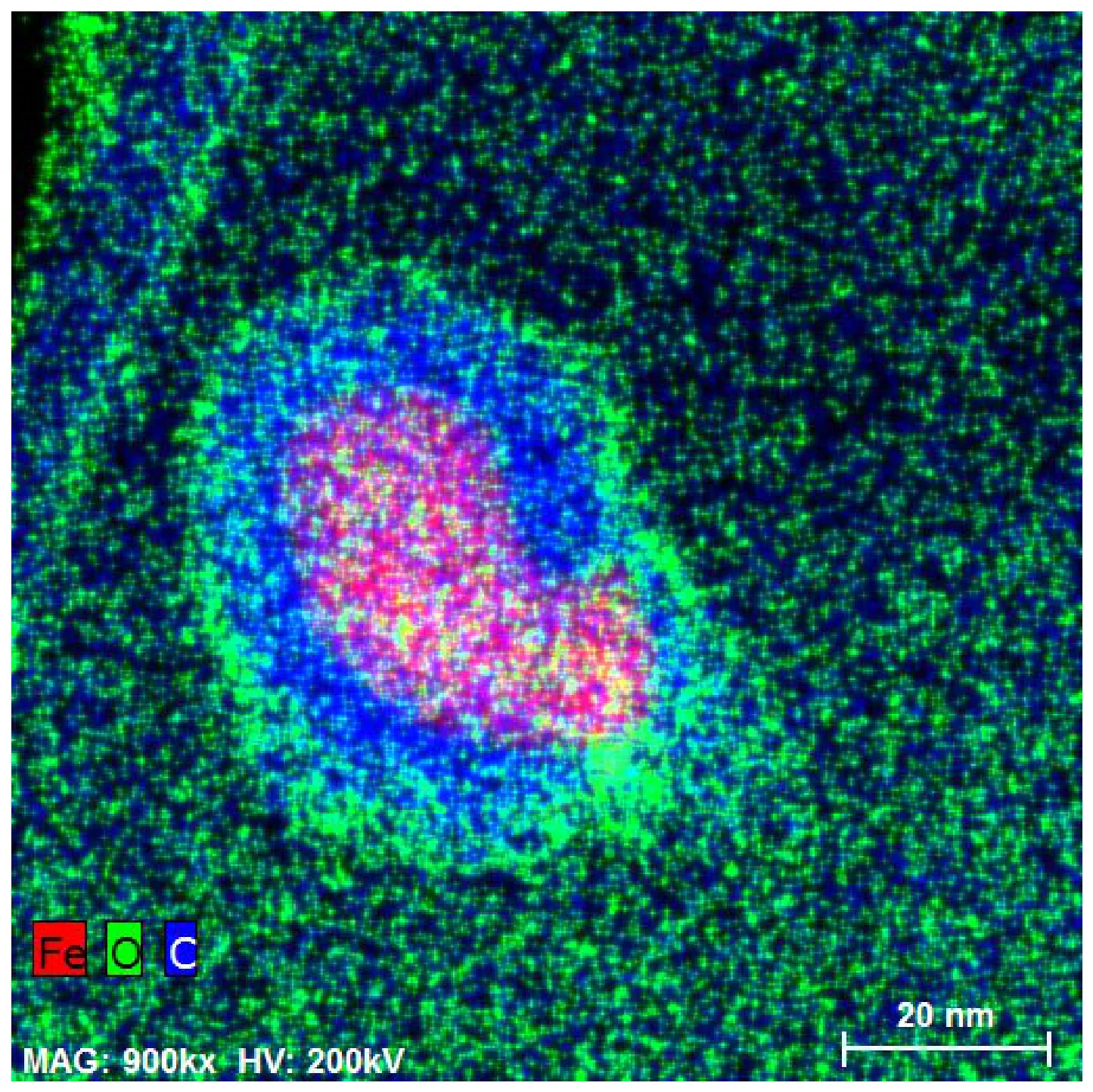
Representative high-magnification Fe/O/C STEM–EDS elemental overlay illustrating a local core–shell-like morphology. Fe-rich signal is concentrated in the central particle-like regions, whereas C- and O-containing signal extends into the surrounding region. The image contains a 20 nm scale bar. Because the rendered elemental channels are qualitative and not common-scale concentration maps, the figure is used as local structural support rather than as a quantitative determination of shell composition, shell thickness, oxidation state, or population-wide architecture.

Figure 10 adds a representative high-magnification Fe/O/C STEM–EDS overlay specifically to address the local core–shell question. The Fe-rich signal is spatially concentrated in the central particle-like regions, while C- and O-containing signal extends more broadly around them. This local elemental differentiation supports describing the displayed object as core–shell-like; however, the qualitative channel scaling does not establish a quantitative shell stoichiometry, a uniform shell thickness, or the crystallographic/oxidation-state identity of the core.

Selected high-resolution fields show dark or high-contrast core-like regions surrounded by lower-contrast layered carbonaceous features. This morphology motivates the term core–shell, but the shell boundary, layer count, and local core phase are not assigned solely from visual contrast. Same-field Fe/C/O maps support Fe association with selected high-contrast regions and a broadly distributed carbon matrix. O_2_ and N_2_ post-annealed fields are described individually.

#### Skeletonized Image-Connectivity Descriptors

The validated cohort contains 12 calibrated FeTCPP bright-field fields: 3 non-annealed, 8 O_2_-annealed, and 1 N_2_-annealed. A total of 1927 retained graph edges were analyzed. Mean tortuosity and mean straightness were robust under the prespecified threshold/pruning sensitivity settings; skeleton-length density and edge-length summaries were moderately sensitive, while orientation concentration, branch density, and cycle density were unstable.

**Figure 11:**
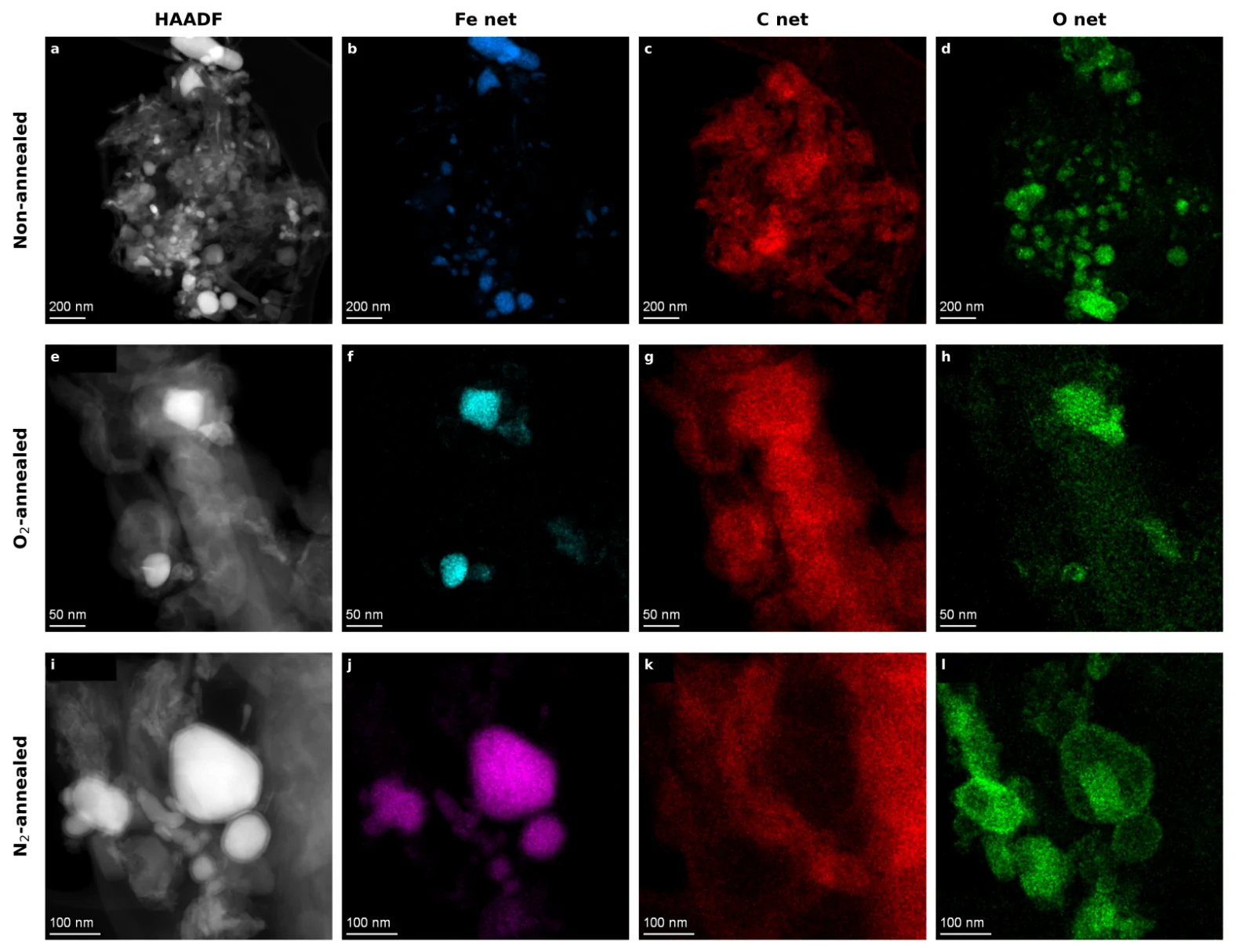
Selected high-angle annular dark-field (HAADF) and qualitative same-acquisition elemental-net images of FeTCPP-derived material initially thermally converted in a vacuum-sealed vessel at 900 °C for 60 min. (**a**) Non-post-annealed HAADF field; (**b**) corresponding Fe net map; (**c**) C net map; (**d**) O net map. (**e**) O_2_-post-annealed HAADF field; (**f**) corresponding Fe net map; (**g**) C net map; (**h**) O net map. (**i**) N_2_-post-annealed HAADF field; (**j**) corresponding Fe net map; (**k**) C net map; (**l**) O net map. Within each row, spatial correspondence between the HAADF image and elemental maps is preserved. Fe signal associated with selected high-contrast regions supports the conservative description “metal-containing region,” while broadly distributed C signal is consistent with a carbonaceous surrounding matrix. Rendered map intensities are qualitative and independently display-scaled. The N_2_ condition is represented by one independent mapped field and is descriptive only.

Figure 11 compares representative FeTCPP HAADF and same-acquisition qualitative elemental-net images across the available post-annealing conditions.

**Figure 12:**
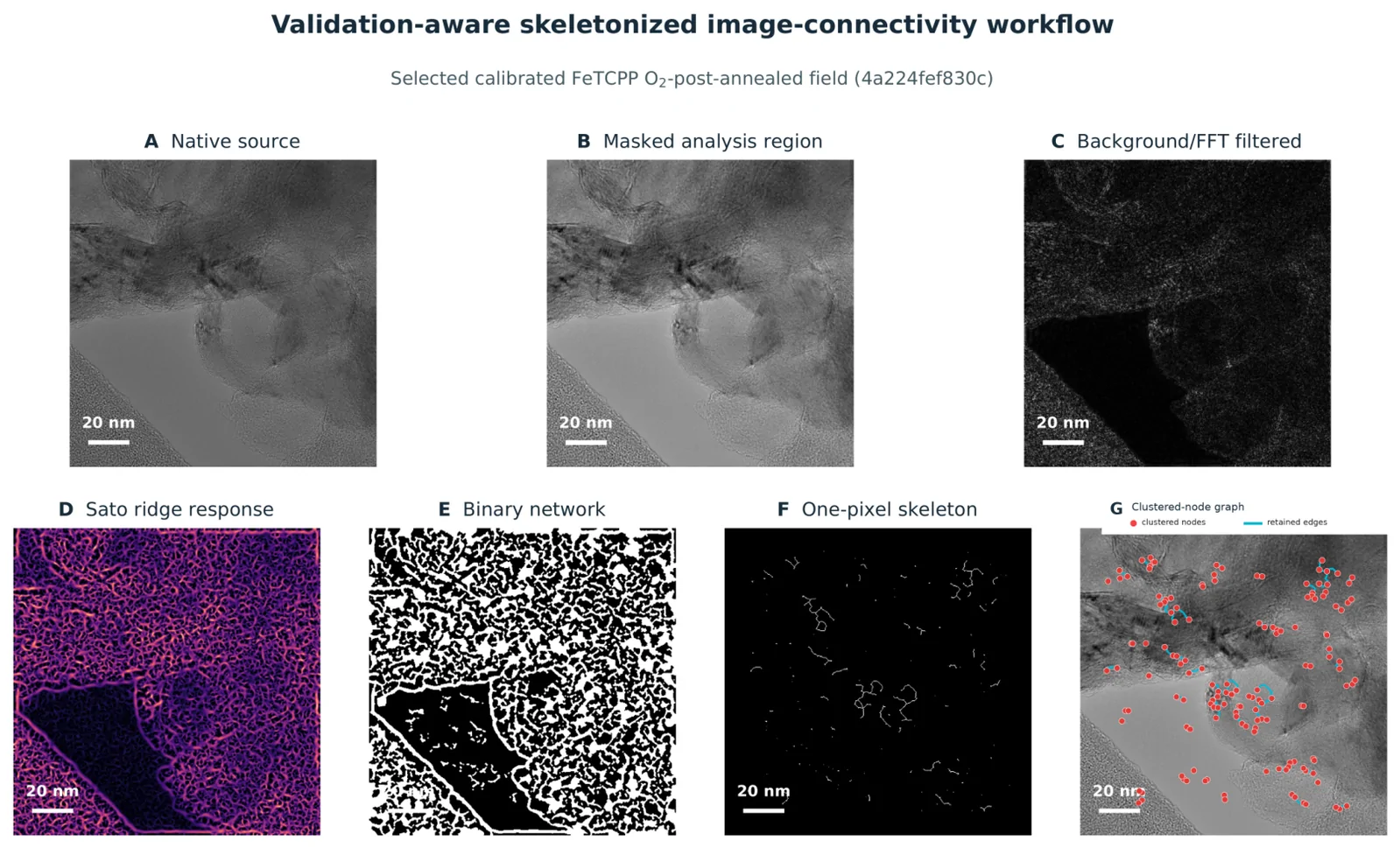
Validation-aware construction of a skeletonized image-connectivity network from a selected calibrated FeTCPP bright-field TEM field. (**A**) Native source field; (**B**) source-coordinate-preserving analysis mask; (**C**) background- and fast-Fourier-transform-filtered field; (**D**) Sato ridge response; (**E**) thresholded binary network; (**F**) one-pixel skeleton; and (**G**) clustered-node graph. In (**G**), red circles mark clustered graph-node regions and cyan line segments mark retained ordered graph edges; the boxed scale bar indicates 20 nm. The workflow provides a reproducible representation of enhanced line-like image contrast rather than a direct map of physical particle junctions or three-dimensional connectivity.

**Figure 13:**
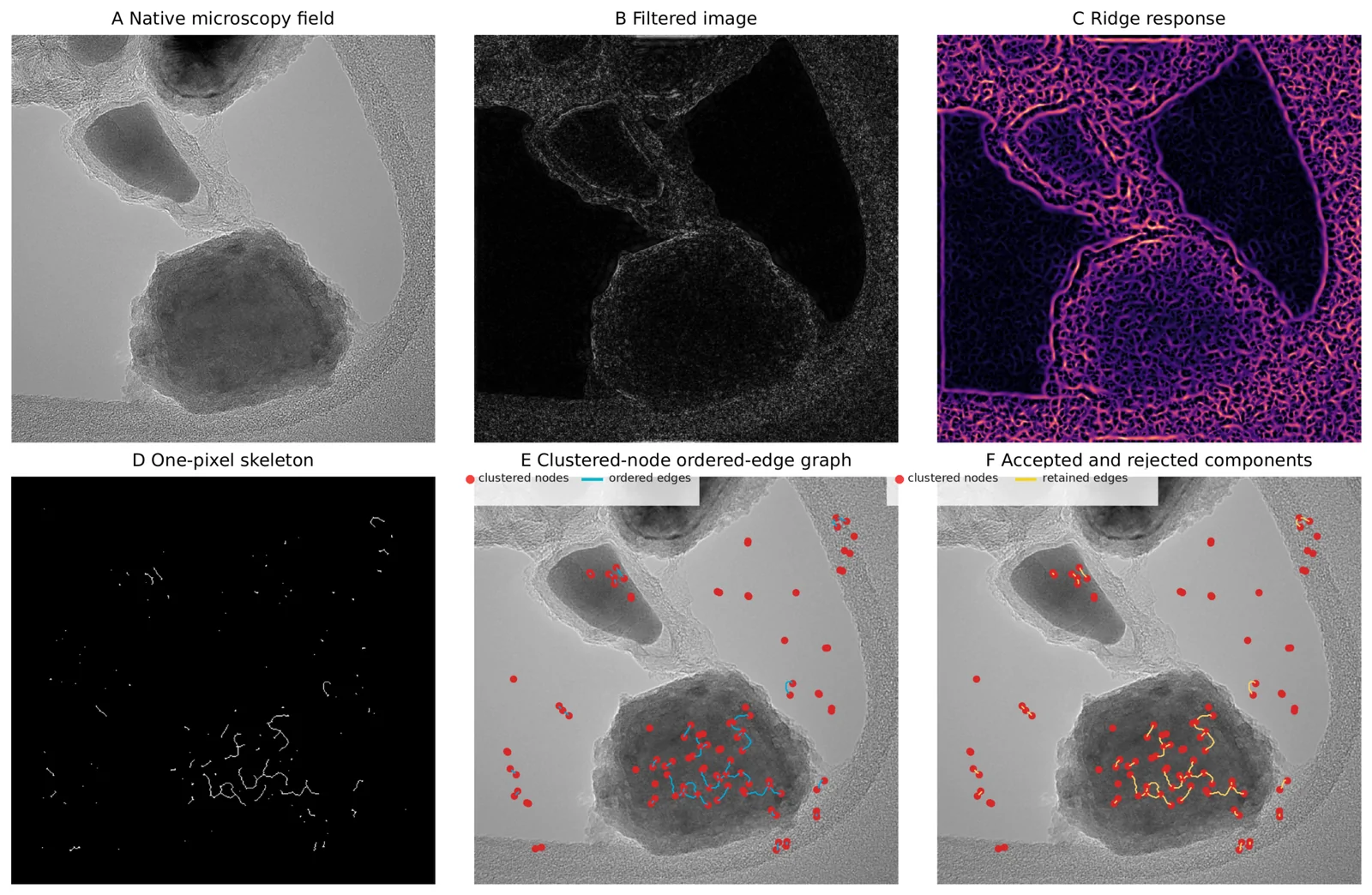
Detailed validation-aware analysis of the native image raster using the revised skeleton-graph toolkit. The annotation/scale-bar band and outer invalid region were excluded before fixed-parameter processing. (**A**) Native microscopy field; (**B**) filtered image; (**C**) ridge-enhanced response; (**D**) one-pixel skeleton; (**E**) clustered-node/ordered-edge graph, where red circles denote clustered node regions and cyan paths denote ordered graph edges; and (**F**) accepted/rejected-component view, where red circles again denote clustered node regions and yellow paths denote ordered edge components retained by the fixed graph-validity criteria. Node candidates not connected by a retained yellow path are not counted as accepted graph edges. The workflow documents the image-processing provenance of the reported two-dimensional connectivity descriptors.

The construction and validation sequence for the two-dimensional skeleton representation is shown in Figure 12, while Figure 13 documents the corresponding detailed toolkit analysis for a representative native raster.

**Figure 14:**
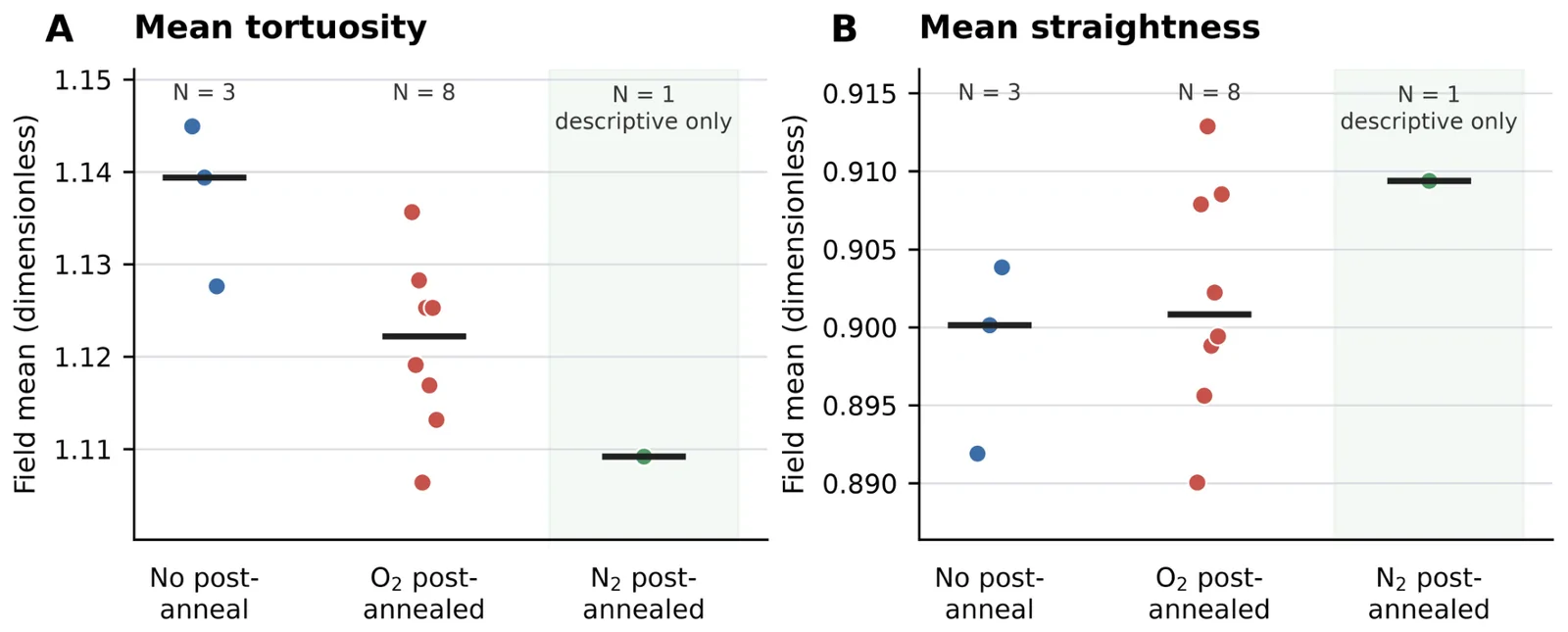
Field-level robust skeletonized image-connectivity descriptors for the calibrated FeTCPP cohort. (**A**) Mean tortuosity and (**B**) mean straightness of graph edges summarized separately for each independent microscopy field. Colored points represent individual fields; black horizontal segments mark group medians. The groups contain non-post-annealed fields (N=3), O_2_-post-annealed fields (N=8), and one N_2_-post-annealed field (N=1). Tortuosity is the retained edge-path length divided by its chord length, whereas straightness is the reciprocal; both are dimensionless descriptors of extracted image-connectivity geometry. These metrics were robust under the prespecified sensitivity settings. The pale N_2_ region and annotation indicate that this group is descriptive only.

Field-level tortuosity and straightness results are summarized graphically in Figure 14 and numerically in Table 6.

### 4.4. Potential Implications

Room-temperature ferromagnetism and spin-dependent magnetic characteristics of carbon nanostructures show promise for non-volatile, energy-efficient graphene-based data storage and spin transport in spintronics. Current research focuses on magnetic nanoparticles of varying phases, sizes, and shapes as high-performance hyperthermia agents. For instance, a high maximum specific loss power value of 215 W/g was recently reported for Fe@C nanocomposites, though this value decreases with oxidation. This performance makes carbon nanocomposites a highly promising candidate for magnetic hyperthermia compared to traditional iron oxides [17]. Concurrently, single-domain iron oxide nanoparticles attract significant attention due to their biocompatibility, biosafety, stable magnetic response, and resistance to oxidation [58,59,60]. Furthermore, carbon-encapsulated iron-containing nanoparticles offer strong potential in catalysis, biomedical delivery, imaging, and electrochemical energy storage, as the carbon shell prevents oxidation and agglomeration while maintaining magnetic and conductive functionalities [3,6,15,16,17,61,62,63,64,65,66,67,68,69,70,71,72,73,74,75]. Finally, iron-phthalocyanine systems have been evaluated in polymer-complex biomedical contexts and magnetically recoverable photocatalytic systems, highlighting distinct application pathways that require dedicated performance and safety testing [76,77].

## 5. Conclusions

Under matched 900 °C, 60 min, and 10 K conditions, FePc exhibited the largest mass-normalized saturation-like magnetization, with $M_{s}=19.66$ emu g^−1^ and $H_{c}=1154$ Oe. FeTCPP showed a lower $M_{s}$ of 8.72 emu g^−1^ but the largest coercivity, $H_{c}=1963$ Oe, whereas FeTPP displayed both the smallest $M_{s}$ (3.26 emu g^−1^) and a much narrower hysteresis loop ($H_{c}=33.72$ Oe). At 300 K, the corresponding $M_{s}$ values decreased to 16.09, 6.89, and 2.38 emu g^−1^ for FePc, FeTCPP, and FeTPP, respectively, while $H_{c}$ decreased to 388.2, 409.9, and 27.99 Oe. Dwell-time comparisons were also non-monotonic: increasing the FeTCPP dwell from 60 to 180 min at 10 K increased $M_{s}$ from 8.72 to 12.60 emu g^−1^ but reduced $H_{c}$ from 1963 to 1198 Oe. These results demonstrate precursor-, temperature-, and processing-dependent differences in magnetization and magnetic reversal; they do not establish a direct correlation between physical nanoparticle size and coercivity.

Beyond size effects, this work elucidates the influence of carbon, nitrogen, and oxygen constituents on the magnetic behavior of iron nanoparticles embedded within distinct carbon matrices, including FePc (FeC_32_H_18_N_8_), FeTCPP (FeC_48_H_30_N_4_O_8_), and FeTPP (FeC_44_H_30_N_4_). Morphological characterization via high-resolution STEM, coupled with an automated segmentation algorithm, revealed pronounced differences among the sampled FePc- and porphyrin-derived fields. Variations in local Fe/C contrast, heteroatom content (N, O), image-derived porosity-like regions, particle-like size distributions, and two-dimensional connectivity descriptors were associated with precursor chemistry and pyrolysis conditions within the available microscopy fields. The ability to quantify these image-derived features, together with their validation and sampling limits, provides a useful comparative framework for rational nanostructure design.

Vacuum-sealed thermal conversion of Pc-, TCPP-, and TPP-based precursors yielded carbon matrices and embedded metal nanocomposites with strongly precursor- and processing-dependent structural and magnetic responses. FePc exhibited the highest mass-normalized magnetization, while FeTCPP demonstrated the broadest low-field hysteresis among Fe-containing samples. Bulk PXRD analysis showed graphite-like carbon together with reflections consistent with candidate Fe- and carbide-related phases, with extracted $d_{002}$ spacing and coherent-domain size ($L_{c}$) varying systematically with precursor type and dwell time. Microscopy and EDS analyses further supported dispersed Fe-rich domains embedded within carbonaceous matrices, while the added representative Fe/O/C STEM–EDS field provides direct local support for a core–shell-like morphology in one observed object. This representative observation is not generalized to a uniform shell thickness, composition, or local phase throughout the specimen population. Additionally, the image-based connectivity analysis provided reproducible two-dimensional descriptors such as tortuosity and straightness, reinforcing the structure–property comparison while remaining distinct from direct measurements of bulk crystallinity, total porosity, or three-dimensional connectivity.

From an application perspective, the robust magnetic properties observed in FePc and FeTCPP systems—particularly their high coercivity and saturation-like magnetization—highlight their potential relevance to biomedical applications such as magnetic hyperthermia and targeted drug delivery. Carbon matrices may also provide useful chemical and interfacial functionality. However, biocompatibility, colloidal behavior, alternating-current (AC)-field heating efficiency, and specific absorption rate (SAR) were not measured in the present work, so these application-specific properties remain to be established experimentally.

The novelty of this work lies in combining a measured metal-free carbon-matrix gauge with a multi-precursor Fe comparison, bulk phase/order analysis, local STEM/EDS evidence, and validation-aware computational morphology within one processing–structure–magnetism study. This approach is important because the carbon host is not magnetically silent, the Fe content and candidate phases can evolve with thermal history, and bulk and local probes do not measure the same structural scale. By analyzing these contributions together rather than attributing the magnetic response to particle size or a single phase alone, the study provides a more defensible basis for selecting precursor chemistry and processing conditions for dispersed magnetic nanocomposites.

Future work will focus on optimizing thermal processing parameters, exploring alternative ligands and dopants, and systematically evaluating nanoparticle performance under varied pyrolysis and post-annealing conditions. Furthermore, the integration of large datasets with advanced analytical and machine learning approaches offers a promising pathway for predictive design and accelerated optimization of magnetic nanomaterials.

A longer-term extension is to translate this precursor-derived carbon-matrix strategy toward compositionally complex and ultimately high-entropy magnetic nanocomposites. Recent high-entropy magnetic materials demonstrate that multielement composition, nanoscale precipitation, and heterogeneous interfaces can substantially modify magnetic response [49,50,51]. The present lower-complexity Fe-based systems are important in that progression because they establish how precursor identity, carbon-host response, thermal history, candidate Fe-containing phases, and local morphology affect magnetism before several magnetic elements are introduced simultaneously. Future mixed-metal macrocyclic precursors or designed precursor mixtures could, therefore, test whether increased compositional complexity yields cooperative magnetic behavior, altered phase stability, or new interfacial effects while retaining nanoscale dispersion within the carbon matrix.

More broadly, combining computational analysis with nanomaterials design across materials science, physics, chemistry, engineering, computer science, and related disciplines can create new opportunities for discovery and optimization of functional nanomaterials. In high-entropy and compositionally complex oxides and alloys, localized atomic interactions and multicomponent design provide routes to room-temperature ferromagnetism and tunable saturation magnetization and coercivity, including reduced-coercivity states relevant to soft-magnetic applications [78]. Conversely, in metal-free carbon-based nanomaterials, pyridinic or pyrrolic nitrogen species and edge-hosted configurations can generate localized magnetic states and provide pinning environments that hinder magnetic reversal, thereby contributing to magnetic anisotropy and coercivity [18,28,79]. These complementary design principles motivate future integration of computation, data analysis, precursor chemistry, and nanoscale characterization to accelerate the design of magnetic nanocomposites across a broader compositional space.

Overall, this work demonstrates that the integration of controlled synthesis, advanced structural and morphological characterization, and data-driven analysis provides a comprehensive strategy for designing next-generation magnetic iron nanoparticles dispersed in different carbon nanostructures and nanocomposites. More broadly, the work is important because it provides an experimentally grounded route for separating host-matrix, processing, structural, and magnetic contributions before application-specific optimization is attempted.

## Figures and Tables

**Table 1:** Magnetic properties of FePc samples. Values are archival common-workflow extractions; digits do not represent propagated experimental uncertainty.

Condition	$M_{s}$ (emu/g)	$H_{c}$ (Oe)	$M_{r}$ (emu/g)	$M_{r}/M_{s}$	Area (Oe emu/g)
10 K, 60 min	19.660	1154	6.290	0.32000	70,369
10 K, N2, 180 min	21.480	842.97	6.750	0.31000	37,193
10 K, 120 min	20.050	786.98	4.490	0.22000	49,616
10 K, N2, 120 min	19.520	776.32	4.240	0.21000	49,736
300 K, 60 min	16.090	388.15	3.390	0.21000	15,648
10 K, 180 min	16.640	689.96	3.140	0.18000	34,670
10 K, N2, 60 min	18.280	732.05	3.320	0.18000	65,068
300 K, 120 min	18.990	289.34	2.290	0.12000	13,462
300 K, 180 min	15.850	286.73	1.670	0.10000	10,259

**Table 2:** Magnetic properties of FeTCPP samples.

Condition	$M_{s}$ (emu/g)	$H_{c}$ (Oe)	$M_{r}$ (emu/g)	$M_{r}/M_{s}$	Area (Oe emu/g)
10 K, 60 min	8.720	1963	3.380	0.38000	42,641
10 K, N2, 60 min	8.380	1897	3.240	0.38000	39,720
10 K, O2, 60 min	8.910	1919	3.420	0.38000	43,980
10 K, 180 min	12.600	1198	2.940	0.23000	39,914
300 K, 60 min	6.890	409.92	1.040	0.15000	7288

**Table 3:** Magnetic properties of the available FeTPP sample.

Condition	$M_{s}$ (emu/g)	$H_{c}$ (Oe)	$M_{r}$ (emu/g)	$M_{r}/M_{s}$	Area (Oe emu/g)
10 K, 60 min	3.260	33.720	0.04500	0.01300	1780
300 K, 60 min	2.380	27.990	0.00940	0.00390	172.61

**Table 4:** Traceable PXRD-derived carbon structural parameters. $L_{c}$ and $L_{a}$ are coherent-domain/order descriptors.

Sample	Dwell (min)	$d_{002}$ (nm)	$L_{c}$ (nm)
FePc	60	0.33799	18.700
FePc	120	0.34030	19.440
FePc	180	0.34146	39.960
FeTCPP	60	0.33458	15.400
FeTCPP	120	0.33383	40.510
FeTCPP	180	0.33496	18.470
TCPP-Ar	10	0.37380	0.96853

**Table 5:** Coherent-domain descriptors from the graphite-like (002) region. FWHM was measured from the raw pattern as follows: linear endpoint baseline, interpolated half-maximum crossings, β converted from degrees to radians, K=0.9, and λ=0.154184 nm. $L_{c}$ is a coherent-domain descriptor.

Sample	Dwell (min)	$2\theta_{002}$ (°)	FWHM (°)	$d_{002}$ (nm)	$L_{c}$ (nm)
FePc	60	26.3696	0.4368	0.33799	18.70
FePc	120	26.1874	0.4198	0.34030	19.44
FePc	180	26.0963	0.2043	0.34146	39.96
FeTCPP	60	26.6429	0.5307	0.33458	15.40
FeTCPP	120	26.7036	0.2017	0.33383	40.51
FeTCPP	180	26.6125	0.4423	0.33496	18.47
FeTCPP (N_2_)	60	26.6733	0.5004	0.33421	16.33
FeTCPP (O_2_)	60	26.7644	0.4813	0.33309	16.98
FeTPP	180	26.6125	0.9056	0.33496	9.02

**Table 6:** Robust field-level skeletonized image-connectivity descriptors for the calibrated FeTCPP bright-field TEM cohort. Values are medians of the field means; *N* denotes independent microscopy fields and *n* denotes retained graph edges. Mean tortuosity and mean straightness were robust to the prespecified parameter perturbations. The N_2_ group contains one independent field and is reported for descriptive completeness only.

Treatment	*N*	*n*	Mean Tortuosity	Mean Straightness
Non-annealed	3	573	1.139	0.900
O_2_-annealed	8	1053	1.122	0.901
N_2_-annealed	1	301	1.109	0.909

**Figure A1:**
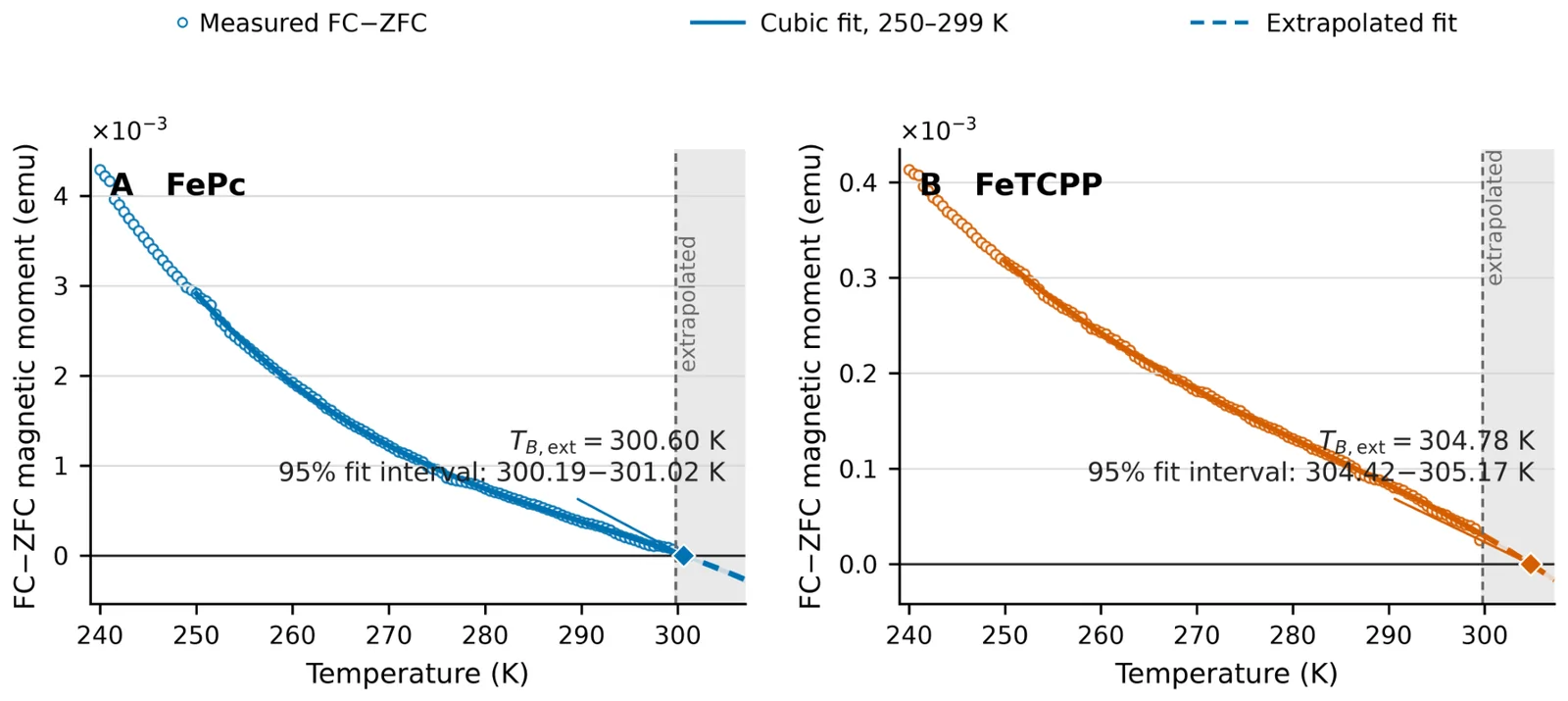
Data-derived extrapolation of the high-temperature ZFC/FC closure measured at 1 kOe. (**A**) FePc and (**B**) FeTCPP. Open symbols show the measured FC−ZFC magnetic-moment difference. Solid curves are cubic fits over the prespecified common 250–299 K interval; dashed portions extend the fitted functions beyond the highest measured temperatures, and the gray regions identify the extrapolation domains. Diamonds mark the zero crossings, yielding $T_{B,\mathrm{ext}}=300.60$ K for FePc and 304.78 K for FeTCPP. The annotated 95% intervals are residual-bootstrap intervals conditional on the cubic model and fit window. The plotted crossings are extrapolated blocking-temperature estimates, not directly observed ZFC maxima.

**Figure A2:**
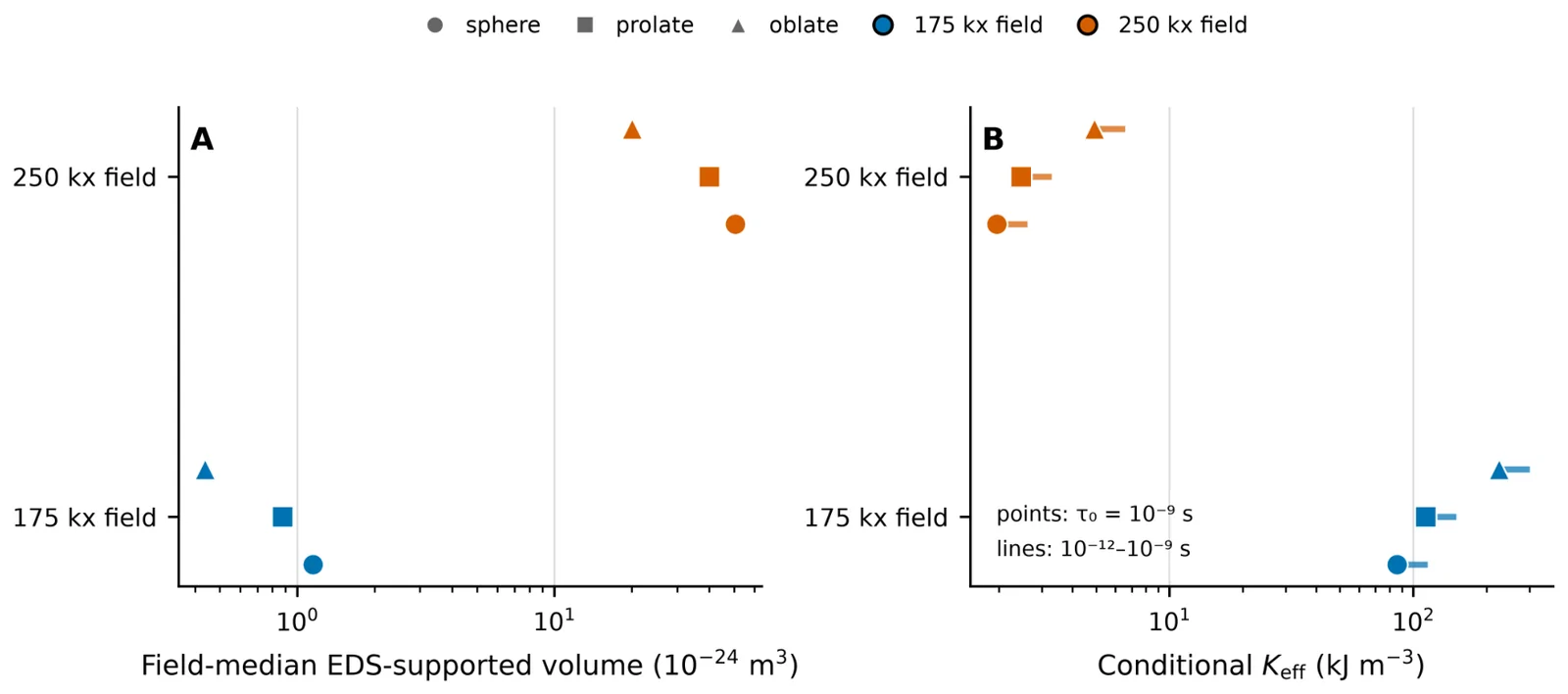
Sensitivity of the FeTCPP volume and $K_{\mathrm{eff}}$ estimates to microscopy field, assumed three-dimensional shape, and attempt time. (**A**) Field-median EDS-supported geometric volumes for spherical, prolate-spheroidal, and oblate-spheroidal depth models. (**B**) Conditional $K_{\mathrm{eff}}$ values calculated with $T_{B,\mathrm{ext}}=304.78$ K and $\tau_{m}\approx 15.0$ s. Symbols show the illustrative $\tau_{0}=10^{-9}$ s scenario; horizontal segments extend to the corresponding value for $\tau_{0}=10^{-12}$ s. The two colors denote independent microscopy fields, while symbol shape denotes the depth model. Accordingly, these are EDS-supported geometric and anisotropy scenarios, not direct measurements of magnetic volume or an intrinsic material constant.

## Data Availability

The datasets presented in this article are not readily available because the data are part of an ongoing study. Requests to access the datasets should be directed to the corresponding author.

## Appendix A. Carbon-Matrix Gauge and Magnetic Interpretation Framework

### Appendix A.1. Decomposition of the Measured Magnetic Response

The magnetic response of a metal-containing carbon specimen may be written schematically as

(A1) $$M_{\mathrm{obs}}\left(H,T\right)=M_{\mathrm{metal}}\left(H,T\right)+M_{\mathrm{matrix}}\left(H,T\right)+M_{\mathrm{interface}}\left(H,T\right)+M_{\mathrm{extrinsic}}\left(H,T\right),$$

where $M_{\mathrm{metal}}$ represents the response of the magnetically active metal-containing domains, $M_{\mathrm{matrix}}$ represents the carbonaceous host, $M_{\mathrm{interface}}$ represents contributions associated with metal–carbon interfaces and interparticle coupling, and $M_{\mathrm{extrinsic}}$ represents the holder, imperfect background subtraction, trace contamination, and other residual contributions. This expression is a conceptual decomposition rather than a procedure for independently extracting the four terms from one hysteresis loop [1,4,5,6,18].

Equation (A1) motivates direct measurements of metal-free precursor-derived matrices. The metal-free matrices, therefore, provide only a contextual reference for the order of magnitude of the carbon-matrix response. They are not subtracted from the metal-containing loops because they were not matched aliquots with identical composition and thermal history; numerical subtraction would introduce an unsupported correction.

### Appendix A.2. Thermally Activated Magnetic Relaxation

For an idealized single-domain magnetic entity with effective anisotropy constant $K_{\mathrm{eff}}$ and magnetically active volume *V*, the anisotropy energy barrier is

(A2) $$\Delta E=K_{\mathrm{eff}}V.$$

Within the Néel–Brown model, the corresponding thermally activated relaxation time is

(A3) $$\tau =\tau_{0}\exp\;\left(\frac{K_{\mathrm{eff}}V}{k_{B}T}\right),$$

where $\tau_{0}$ is the attempt time and $k_{B}$ is the Boltzmann constant. Here, τ is the characteristic thermally activated magnetic-relaxation time of the modeled entity, whereas $\tau_{0}$ is the inverse attempt frequency that sets the microscopic prefactor of the model. The measurement probes a blocking condition when τ becomes comparable to the effective observation time $\tau_{m}$. In this study, the archived PPMS point-level record reports a VSM averaging/observation time of approximately $\tau_{m}=15.0$ s. This quantity describes the instrumental observation window used in the illustrative sensitivity analysis; it is not a fitted magnetic-relaxation time of the sample. No frequency-dependent susceptibility or relaxation series was available to fit $\tau_{0}$; consequently, $\tau_{0}$ was not calculated or measured. It was varied from $10^{-12}$ to $10^{-9}$ s, and $10^{-9}$ s was used only as an illustrative assumed reference. An operational blocking temperature associated with a measurement time $\tau_{m}$ is then

(A4) $$T_{B}=\frac{K_{\mathrm{eff}}V}{k_{B}\ln\left(\tau_{m}/\tau_{0}\right)}.$$

Equations (A2)–(A4) explain why a reported blocking-like temperature is not solely a material constant. It can depend on magnetic volume, anisotropy, interactions, the size distribution, the applied field, and the timescale of the measurement [11,12,13,14,64,80]. For this reason, ZFC/FC irreversibility is described as evidence of slow or blocked magnetic relaxation under the specified protocol, rather than as unique proof of one magnetic phase.

### Appendix A.3. Operational Hysteresis-Loop Descriptors

The loop parameters used in this work are defined operationally by

(A5) $$M\left(H_{c}\right)=0,\;M_{r}=M\left(H=0\right),\;R=\frac{M_{r}}{M_{s}},$$

where $H_{c}$ is the coercive field, $M_{r}$ is the branch remanence, $M_{s}$ is the reported high-field saturation-like magnetization, and *R* is the remanence ratio. The hysteresis-loop area is

(A6) $$A_{\mathrm{loop}}=\oint M\;dH,$$

and is a protocol-dependent measure of hysteretic loss.

These descriptors should be interpreted together. In particular, a large apparent coercive field in a loop with extremely small $M_{s}$, $M_{r}$, and area can result from a weak or background-sensitive zero crossing and does not necessarily indicate a high-energy-product magnetic material.

### Appendix A.4. Previously Reported Metal-Free Carbon Matrices

The complete metal-free carbon-matrix series below was reported in our previous article [30]. The specimens were prepared using the earlier 10 min primary thermal-conversion protocol and selected O_2_, N_2_, and Ar post-annealing treatments. The tables are reproduced here to establish the magnitude and variability of the host response used to motivate the present metal-containing study.

#### Appendix A.4.1. Reported Room-Temperature Magnetism in Carbon Matrices

Weak magnetic signals and hysteretic behavior have been reported in defect-rich, edge-rich, heteroatom-functionalized, and mixed-$sp^{2}/sp^{3}$ carbon materials, including graphite-related systems and functionalized graphene derivatives [41,79,81,82,83]. A notable graphite-related example is the reported ferromagnetism of a graphite nodule from the Canyon Diablo meteorite [29]. Proposed mechanisms include localized electronic states associated with defects, zigzag edges, vacancies, and heteroatoms coupled through the carbon π-electron system [84,85]. These literature reports provide relevant context for the present precursor-derived carbon matrices, but they do not establish the microscopic origin of the archived matrix signals measured here. Magnetic measurements on nominally metal-free carbon are particularly sensitive to trace Fe-containing contamination, residual precursor species, sample-holder contributions, and background subtraction. Because no trace-element assay with sufficient sensitivity was available for the archived Pc-, TPP-, and TCPP-derived matrices, their measured hysteretic components are used here as an empirical host-response scale rather than as proof of intrinsic ferromagnetism in pristine, defect-free graphite. The present data, therefore, do not distinguish uniquely between defect- or heteroatom-associated carbon magnetism and possible extrinsic contributions.

For the present comparison, the important result is quantitative rather than mechanistic: the magnetization of the principal FePc- and FeTCPP-derived specimens exceeds the characteristic metal-free matrix response by orders of magnitude. The matrix curves are not numerically subtracted from the Fe-containing specimens because the samples are not matched in composition, conversion time, or recovered mass. Literature on defect- and heteroatom-associated magnetism nevertheless motivates future controlled studies in which trace-element analysis, defect density, and $sp^{2}/sp^{3}$ bonding are measured independently before assigning a microscopic carbon-magnetism mechanism [8,86].

#### Appendix A.4.2. Pc-Derived Carbon Matrix

The Pc-derived matrix shows small but measurable hysteretic components. Although the measured response depends on temperature and atmosphere, the absolute magnetization remains much smaller than that of the principal FePc specimens considered in the present study. The comparison, therefore, supports a substantial metal-containing contribution in FePc, while not requiring the Pc-derived matrix to be assumed perfectly diamagnetic. The complete Pc host–response values are listed in Table A1.

**Table A1:** Previously reported magnetic properties of Pc-derived carbon matrices after the 10 min primary conversion series [30]. The values define the Pc host–response scale for the present study.

Condition	$M_{s}$ (emu/g)	$H_{c}$ (Oe)	$M_{r}$ (emu/g)	$M_{r}/M_{s}$	Area (Oe emu/g)
300 K	0.02300	99.24	0.001500	0.06700	10.45
300 K, O_2_	0.01600	75.82	0.001000	0.06100	7.440
10 K	0.06100	309.9	0.003700	0.06100	48.24
10 K, Ar	0.04472	88.64	0.002400	0.05500	22.40
300 K, N_2_	0.01193	98.32	0.0006300	0.05300	5.380
300 K, Ar	0.01556	77.07	0.0007600	0.04900	4.510
10 K, N_2_	0.02710	178.2	0.001300	0.04800	21.89
10 K, O_2_	0.04113	199.3	0.001900	0.04600	16.08

#### Appendix A.4.3. TPP-Derived Carbon Matrix

The TPP-derived matrix generally shows small remanence and remanence ratios, although the apparent coercive field varies considerably among measurement conditions. This host scale is especially relevant for weak FeTPP- and CuTPP-family loops, for which the matrix and background terms may represent a larger fraction of the total response than in FePc or FeTCPP. The full TPP matrix dataset is summarized in Table A2.

**Table A2:** Previously reported magnetic properties of TPP-derived carbon matrices after the 10 min primary conversion series [30].

Condition	$M_{s}$ (emu/g)	$H_{c}$ (Oe)	$M_{r}$ (emu/g)	$M_{r}/M_{s}$	Area (Oe emu/g)
10 K, N_2_	0.005500	254.1	0.0006600	0.1100	6.420
10 K	0.01500	300.0	0.0004400	0.02800	4.710
10 K, Ar	0.01000	210.4	0.0003000	0.02800	6.140
10 K, O_2_	0.02600	210.4	0.0005500	0.02000	16.89
300 K, O_2_	0.007500	177.2	0.0001300	0.01700	5.590
300 K, N_2_	0.01100	776.2	0.0001600	0.01400	1.180
300 K, Ar	0.01300	375.1	0.0001600	0.01200	5.530
300 K	0.01500	298.3	0.0001200	0.008100	0.09400

#### Appendix A.4.4. TCPP-Derived Carbon Matrix

The TCPP-derived records show the broadest variation among the three metal-free families. Several entries have relatively large $M_{r}/M_{s}$ values despite very small absolute magnetization. The corresponding TCPP matrix values are reported in Table A3.

**Table A3:** Previously reported magnetic properties of TCPP-derived carbon matrices after the 10 min primary conversion series [30].

Condition	$M_{s}$ (emu/g)	$H_{c}$ (Oe)	$M_{r}$ (emu/g)	$M_{r}/M_{s}$	Area (Oe emu/g)
300 K, O_2_	0.001490	488.1	0.0005900	0.3986	3.129
10 K	0.002510	1063	0.0008500	0.3381	13.78
10 K	0.007220	1785	0.0005100	0.07040	6.828
10 K, O_2_	0.01985	532.5	0.001160	0.05840	12.37
10 K	0.01075	386.3	0.0005800	0.05430	6.655
10 K, Ar	0.002930	1363	0.0001600	0.05400	4.706
10 K, N_2_	0.01394	410.5	0.0005900	0.04240	7.922
300 K	0.01579	1230	0.0004300	0.02750	5.982
300 K, Ar	0.01246	2319	0.0003000	0.02440	11.95
300 K	0.01568	464.0	0.0002900	0.01840	0.9260
300 K	0.03227	943.8	0.0005100	0.01580	10.17
300 K, N_2_	0.01332	698.1	0.0001100	0.008400	3.259

### Appendix A.5. Balance Between the Prior Matrix Study and the Present Nanoparticle Study

The matrix and nanoparticle datasets answer related but distinct questions. The previous work established that metal-free Pc-, TPP-, and TCPP-derived carbon can exhibit small, atmosphere-sensitive magnetic loops after short conversion [30]. The present study examines how the response changes when transition-metal-containing precursors are converted for substantially longer dwell times and form heterogeneous metal-containing regions within carbonaceous matrices.

Across Table A1, Table A2 and Table A3, most metal-free values of $M_{s}$ are of order $10^{-3}$–$10^{-2}$ emu g^−1^, and the largest listed value remains below 0.1 emu g^−1^. By comparison, the principal Fe-containing specimens considered in the main text exhibit magnetizations of order 1–20 emu g^−1^. The FePc and FeTCPP responses, therefore, exceed the characteristic metal-free host scale by approximately two to four orders of magnitude under the displayed conditions.

This magnitude difference supports a substantial contribution from the metal-containing domains. It does not justify direct subtraction of an unmatched matrix loop, because the previous and present specimens differ in conversion time, composition, thermal history, mass normalization, and potential residual phases.

Coercivity does not show the same simple magnitude separation. A large zero-crossing field can occur in a loop with very small absolute moment and may be sensitive to background subtraction, field spacing, or branch interpolation. Comparisons between matrix and metal-containing materials should consequently consider $M_{s}$, $M_{r}$, $M_{r}/M_{s}$, and loop area together rather than ranking magnetic strength from $H_{c}$ alone.

### Appendix A.6. Carbon Ordering and Post-Annealing Context

The graphite-like (002) reflection provides an interlayer spacing $d_{002}$ and an out-of-plane coherent-domain descriptor $L_{c}$. A reproducible in-plane $L_{a}$ estimate additionally requires a traceable (100) or (101) reflection and its corrected peak width [52,53,87,88,89,90]. These PXRD quantities describe average carbon ordering and coherent diffraction domains.

The O_2_, N_2_, and Ar post-annealing records in the previous matrix study show that processing atmosphere can accompany changes in the measured magnetic response.

## Appendix B. Magnetic Anisotropy Calculation Framework

The effective magnetic anisotropy can, in principle, be estimated from an independently determined anisotropy field $H_{k}$ according to

(A7) $$K_{\mathrm{eff}}\approx \frac{1}{2}\mu_{0}M_{s}H_{k},$$

where $M_{s}$ must be expressed as a volumetric magnetization in A m^−1^. This route was not applied here because no defensible $H_{k}$ was recovered and the measured $M_{s}$ values are normalized by total specimen mass. In particular, the coercive field was not substituted for $H_{k}$, and values in emu g^−1^ were not inserted directly into Equation (A7).

For a thermally activated single-barrier scenario, the Néel–Brown relation gives

(A8) $$\tau_{m}=\tau_{0}\exp\;\left(\frac{K_{\mathrm{eff}}V}{k_{B}T_{B,\mathrm{ext}}}\right),$$

and, therefore,

(A9) $$E_{a}=K_{\mathrm{eff}}V=k_{B}T_{B,\mathrm{ext}}\ln\;\left(\frac{\tau_{m}}{\tau_{0}}\right),\;K_{\mathrm{eff}}=\frac{k_{B}T_{B,\mathrm{ext}}}{V}\ln\;\left(\frac{\tau_{m}}{\tau_{0}}\right).$$

Here, $T_{B,\mathrm{ext}}$ denotes an extrapolated blocking-temperature estimate, *V* is the assumed magnetic volume of the relaxing entity, $\tau_{m}$ is the effective observation time, and $\tau_{0}$ is the magnetic attempt time. Equation (A9) is consequently model dependent and does not establish that the specimens contain noninteracting, monodisperse, single-domain particles.

### Appendix B.1. Data-Derived Blocking-Temperature Extrapolation

The blocking-temperature analysis used the 1 kOe ZFC/FC measurements. The FC and ZFC branches were interpolated to a common 0.5 K temperature grid, and the high-temperature difference $\Delta m\left(T\right)=m_{\mathrm{FC}}\left(T\right)-m_{\mathrm{ZFC}}\left(T\right)$ was fitted with a cubic polynomial over the common 250–299 K interval. The extrapolated closure Δm=0 gave

(A10) $$T_{B,\mathrm{ext}}^{\mathrm{FePc}}=300.60\;K,\;T_{B,\mathrm{ext}}^{\mathrm{FeTCPP}}=304.78\;K.$$

The highest measured temperatures were 299.808 K for FePc and 299.814 K for FeTCPP. Thus, the closures lie 0.80 and 4.96 K, respectively, outside the measured range and are not observed ZFC maxima. Residual bootstrap resampling of the specified cubic fit gave conditional 95% fit intervals of 300.19–301.02 K for FePc and 304.42–305.17 K for FeTCPP. These intervals quantify residual uncertainty for the stated model and fit window; they do not include the full systematic uncertainty associated with selecting a polynomial form or extrapolation interval. The measured high-temperature FC–ZFC differences, fitted extrapolations, and conditional closure estimates are displayed in Figure A1.

The point-level PPMS records report a VSM averaging time of approximately $\tau_{m}\;=\;15.0$ s for both datasets; the approximately 14.99 s median separation between adjacent high-temperature records provides an independent timing check. This quantity is used here only as an instrumental observation-time estimate for the illustrative Néel–Brown sensitivity calculation; it is not a fitted sample magnetic-relaxation time. No frequency-dependent AC-susceptibility or magnetic-relaxation series was recovered, so $\tau_{0}$ was not measured. We, therefore, used an explicit sensitivity interval $10^{-12}\leq \tau_{0}\leq 10^{-9}$ s, with $10^{-9}$ s as the illustrative reference scenario. Equation (A9) then gives extrapolation-based energy barriers of 0.6069–0.7859 eV for FePc and 0.6154–0.7968 eV for FeTCPP over the assumed $\tau_{0}$ interval. These are energy-barrier scenarios rather than independent measurements of an intrinsic anisotropy constant.

### Appendix B.2. EDS-Supported FeTCPP Volume and Conditional $K_{\mathrm{eff}}$

A numerical $K_{\mathrm{eff}}$ additionally requires the magnetically active volume. No calibrated, condition-matched FePc magnetic-domain volume was recovered; FePc $K_{\mathrm{eff}}$, therefore, remains undetermined. For FeTCPP, two high-magnification, same-acquisition HAADF/Fe-net map sets from the nominally matched non-post-annealed 900 °C/60 min condition were used to construct EDS-supported geometric-volume scenarios. A common processing rule was then applied to the blue-encoded Fe-net signal: Gaussian smoothing, Otsu thresholding, physical morphological closing, minimum-size and solidity filtering, and rejection of components contacting invalid boundaries. Fourteen components were retained in the 175 kx field and four in the 250 kx field. The respective field-median Fe-supported equivalent diameters were 12.74 and 45.54 nm.

Because only two projected axes are observed, three depth assumptions were evaluated. The sphere used $V=\pi d_{\mathrm{eq}}^{3}/6$; the prolate spheroid used $V=\pi d_{\mathrm{maj}}d_{\min}^{2}/6$; and the oblate scenario used an assumed third axis of $0.5d_{\min}$, giving $V=\pi d_{\mathrm{maj}}d_{\min}^{2}/12$. The resulting field-median volumes and conditional anisotropy ranges are summarized in Table A4.

**Table A4:** Field-level EDS-supported FeTCPP geometric-volume and conditional anisotropy scenarios. *n* is the number of retained two-dimensional Fe-net components, not the number of independent synthesis replicates. Volume ranges span the three depth models. $K_{\mathrm{eff}}$ ranges combine those depth models with the assumed $10^{-12}$–$10^{-9}$ s attempt-time interval.

Field	*n*	Median $d_{\mathrm{eq}}$ (nm)	Volume Range ($10^{-24}$ m^3^)	$K_{\mathrm{eff}}$ Range (kJ m^−3^)
175 kx	14	12.74	0.4385–1.151	85.68–291.6
250 kx	4	45.54	20.03–50.46	1.954–6.386

The sensitivity of these field- and shape-dependent volume and anisotropy scenarios is shown in Figure A2.

The two fields yield strongly separated volume distributions and, consequently, strongly separated $K_{\mathrm{eff}}$ scenarios. Pooling their components would conceal this field dependence and would treat image components as independent experimental replicates. We, therefore, report the six field/shape combinations explicitly and do not select a preferred FeTCPP $K_{\mathrm{eff}}$. The rendered EDS maps support localization of Fe-containing image regions, but independent display scaling prevents quantitative compositional thresholding. The dominant limitation is, therefore, the magnetic-volume assignment, followed by the unmeasured attempt time. Frequency-dependent AC susceptibility together with registered, raw-count HAADF/EDS measurements from the same synthesis batch would most directly reduce these uncertainties.

## Appendix C. Computational Toolkits

Custom computational workflows were used to extract magnetic, structural, and image-derived descriptors. Each workflow applies a defined sequence of preprocessing, feature extraction, quality control, and summary operations rather than relying on manual visual selection alone.

For magnetometry, the measured magnetic moment is first normalized by the total specimen mass to obtain the mass magnetization,

(A11) $$M\left(H\right)=\frac{m(H)}{m_{\mathrm{sample}}},$$

where m(H) is the field-dependent magnetic moment and $m_{\mathrm{sample}}$ is the measured sample mass. The ascending and descending field branches are separated according to the acquisition order. The coercive fields are then obtained from the zero crossings of M(H) on the two branches, using local linear interpolation between adjacent field points that bracket M=0. The reported coercivity is calculated from the magnitudes of the two branch crossings,

(A12) $$H_{c}=\frac{|H_{c,+}\left|\;+\;\right|H_{c,-}|}{2}.$$

The remanent magnetization is obtained analogously by interpolating each branch to H=0, and the reported remanence is

(A13) $$M_{r}=\frac{|M_{r,+}\left|\;+\;\right|M_{r,-}|}{2}.$$

The high-field magnetization $M_{s}$ is estimated from the measured high-field region using the same selection rule for all samples. The ratio $M_{r}/M_{s}$ is then used as a dimensionless descriptor of loop squareness. Full-field and low-field views are generated from the same normalized data so that saturation-like behavior, coercive width, and remanence can be inspected without changing the underlying measurements.

For PXRD analysis, the measured intensity I(2θ) is first corrected for slowly varying background contributions. Candidate diffraction maxima are identified from local intensity maxima that exceed a fixed prominence or signal-to-background threshold. Each retained reflection is fitted within a bounded angular window using an analytical peak profile together with a local background term. The fitted peak center provides the Bragg angle, while the fitted full width at half maximum is converted to radians and corrected for instrumental broadening when a reference width is available. Interplanar spacing is calculated from Bragg’s law,

(A14) $$d_{hkl}=\frac{\lambda }{2\sin\theta_{hkl}},$$

and the coherent-domain dimension associated with a selected reflection is estimated using the Scherrer relation,

(A15) $$L_{hkl}=\frac{K\lambda }{\beta_{hkl}\cos\theta_{hkl}},$$

where *K* is the selected shape factor, λ is the X-ray wavelength, and $\beta_{hkl}$ is the corrected peak width. For graphitic carbon, the (002) reflection is used to estimate $d_{002}$ and the stacking-direction coherent-domain dimension $L_{c}$. An in-plane dimension $L_{a}$ is reported only when an appropriate in-plane reflection and a reproducible width-extraction rule are available. Peak assignments are compared with candidate reference patterns, but phase labels remain bulk-sample interpretations and are not transferred automatically to individual microscopy domains.

Local periodicity is examined using a windowed Fourier-transform procedure. The image is divided into overlapping spatial patches, and each patch is multiplied by a smooth window to reduce edge discontinuities. The two-dimensional Fourier power spectrum is then evaluated within a prespecified spatial-frequency band. The dominant radial frequency is converted to a local spacing through

(A16) $$d=\frac{1}{q},$$

after accounting for the physical pixel calibration, where *q* is the selected spatial frequency. The azimuthal location of the Fourier maximum provides an axial orientation modulo $180^{\circ }$. Angular concentration around the dominant direction is used as a coherence descriptor. These local Fourier outputs are conditional on the selected patch size, overlap, and frequency range; they do not independently establish crystallographic phase or count individual atomic planes.

The skeleton-network workflow is designed to characterize line-like contrast in microscopy images. After grayscale normalization and background correction, line-like structures are enhanced with a multiscale ridge filter based on the local Hessian eigenvalues. The ridge-response image is converted to a binary network using a fixed local-threshold rule. Small isolated objects and small holes are removed, short gaps are closed morphologically, and the resulting network is thinned to a one-pixel-wide skeleton. Short terminal spurs are pruned using a fixed endpoint-removal depth.

Graph construction is performed only after skeletonization. Skeleton pixels with three or more local neighbors are first identified as candidate branch pixels. Spatially adjacent branch pixels are clustered into a single node region so that one physical branch neighborhood is not counted repeatedly as multiple junction pixels. Endpoints are represented as degree-one nodes. After node regions are removed from the skeleton, each remaining connected path is traced in pixel order and retained as a graph edge only when it connects exactly two valid nodes. Components with malformed attachment, duplicate ownership, zero chord length, or ambiguous ordering are rejected and logged rather than forced into the graph. Isolated closed components are retained separately.

For each accepted edge, the path length is computed from the ordered pixel chain using unit weights for horizontal and vertical steps and $\sqrt{2}$ weights for diagonal steps. The chord length is the Euclidean distance between the two associated node centroids. Tortuosity and straightness are then defined as

(A17) $$\tau =\frac{L_{\mathrm{path}}}{L_{\mathrm{chord}}},\;S=\frac{L_{\mathrm{chord}}}{L_{\mathrm{path}}}=\tau^{-1}.$$

Valid edges, therefore, satisfy τ≥1 and 0≤S≤1, within numerical tolerance. Edge orientation is treated as an axial quantity over $0^{\circ }$–$180^{\circ }$, so circular rather than ordinary linear statistics are used.

To avoid treating thousands of graph edges as independent experimental replicates, quantitative comparisons are performed at the image-field level. For field-balanced edge distributions, every edge *j* from field *i* receives weight

(A18) $$w_{ij}=\frac{1}{Nn_{i}},$$

where *N* is the number of independent fields in the displayed group and $n_{i}$ is the number of accepted edges in field *i*. Thus, each image contributes equal total weight regardless of skeleton density. The revised implementation reports clustered graph nodes, ordered edges, tortuosity, straightness, and sensitivity-qualified field summaries.

## References

[1] Singamaneni S., Bliznyuk V.N., Binek C., Tsymbal E.Y. (2011). Magnetic Nanoparticles: Recent Advances in Synthesis, Self-Assembly and Applications. J. Mater. Chem..

[2] Skumryev V., Stoyanov S., Zhang Y., Hadjipanayis G., Givord D., Nogués J. (2003). Beating the Superparamagnetic Limit with Exchange Bias. Nature.

[3] Issa B., Obaidat I.M., Albiss B.A., Haik Y. (2013). Magnetic Nanoparticles: Surface Effects and Properties Related to Biomedicine Applications. Int. J. Mol. Sci..

[4] Kodama R.H. (1999). Magnetic Nanoparticles. J. Magn. Magn. Mater..

[5] Leslie-Pelecky D.L., Rieke R.D. (1996). Magnetic Properties of Nanostructured Materials. Chem. Mater..

[6] Lu A.H., Salabas E.L., Schüth F. (2007). Magnetic Nanoparticles: Synthesis, Protection, Functionalization, and Application. Angew. Chem. Int. Ed..

[7] Alagiri M., Muthamizhchelvan C., Ponnusamy S. (2011). Structural and Magnetic Properties of Iron, Cobalt and Nickel Nanoparticles. Synth. Met..

[8] Gyulasaryan A.T., Castillo K.A., Bernal O.O., Kocharian A.N., Sisakyan N., Chilingaryan G.K., Veligzhanin A.A., Gray J.L., Sharoyan E.G., Manukyan A.S. (2021). Synthesis and Structure of Fe–Fe3O4 Nanoparticles with “Core–Shell” Architecture Capsulated in a Graphite-Like Carbon Matrix. J. Contemp. Phys. (Armenian Acad. Sci.).

[9] Manukyan A., Elsukova A., Mirzakhanyan A., Gyulasaryan H., Kocharian A., Sulyanov S., Spasova M., Römer F., Farle M., Sharoyan E. (2018). Structure and Size Dependence of the Magnetic Properties of Ni@C Nanocomposites. J. Magn. Magn. Mater..

[10] Manukyan A., Avakyan L., Elsukova A., Zubavichus Y., Sulyanov S., Mirzakhanyan A., Kolpacheva N., Spasova M., Kocharian A., Farle M. (2018). Formation of Nickel Nanoparticles and Magnetic Matrix in Nickel Phthalocyanine by Doping with Potassium. Mater. Chem. Phys..

[11] Bean C.P., Livingston J.D. (1959). Superparamagnetism. J. Appl. Phys..

[12] Stoner E.C., Wohlfarth E.P. (1948). A Mechanism of Magnetic Hysteresis in Heterogeneous Alloys. Philos. Trans. R. Soc. Lond. Ser. A Math. Phys. Sci..

[13] Kneller E.F., Luborsky F.E. (1963). Particle Size Dependence of Coercivity and Remanence of Single-Domain Particles. J. Appl. Phys..

[14] Batlle X., Labarta A. (2002). Finite-Size Effects in Fine Particles: Magnetic and Transport Properties. J. Phys. D Appl. Phys..

[15] Nemati Z., Alonso J., Khurshid H., Phan M.H., Srikanth H. (2016). Core/Shell Iron/Iron Oxide Nanoparticles: Are They Promising for Magnetic Hyperthermia?. RSC Adv..

[16] Lak A., Niculaes D., Anyfantis G.C., Bertoni G., Barthel M.J., Marras S., Cassani M., Nitti S., Athanassiou A., Giannini C. (2016). Facile transformation of FeO/Fe3O4 core-shell nanocubes to Fe3O4 via magnetic stimulation. Sci. Rep..

[17] Sargsyan A.H., Kocharian A.N., Gyulasaryan H.T., Makridis A., Bernal O.O., Gray J.L., Angelakeris M., Mukasyan A.S., Manukyan A.S. (2025). Core–Shell Fe-Based Nanoparticles in a Carbon Matrix: Synthesis and Magnetic Properties. J. Mater. Sci..

[18] Błoński P., Tuček J., Sofer Z., Mazánek V., Petr M., Pumera M., Otyepka M., Zbořil R. (2017). Doping with Graphitic Nitrogen Triggers Ferromagnetism in Graphene. J. Am. Chem. Soc..

[19] Esquinazi P., Setzer A., Höhne R., Semmelhack C., Kopelevich Y., Spemann D., Butz T., Kohlstrunk B., Lösche M. (2002). Ferromagnetism in Oriented Graphite Samples. Phys. Rev. B.

[20] Červenka J., Katsnelson M.I., Flipse C.F.J. (2009). Room-Temperature Ferromagnetism in Graphite Driven by Two-Dimensional Networks of Point Defects. Nat. Phys..

[21] Nair R.R., Sepioni M., Tsai I., Lehtinen O., Keinonen J., Krasheninnikov A.V., Thomson T., Geim A.K., Grigorieva I.V. (2012). Spin-Half Paramagnetism in Graphene Induced by Point Defects. Nat. Phys..

[22] Yazyev O.V., Helm L. (2007). Defect-Induced Magnetism in Graphene. Phys. Rev. B.

[23] Palacios J.J., Fernández-Rossier J., Brey L. (2008). Vacancy-Induced Magnetism in Graphene and Graphene Ribbons. Phys. Rev. B.

[24] Ugeda M.M., Brihuega I., Guinea F., Gómez-Rodríguez J.M. (2010). Missing Atom as a Source of Carbon Magnetism. Phys. Rev. Lett..

[25] Sepioni M., Nair R.R., Rablen S., Narayanan J., Tuna F., Winpenny R., Geim A.K., Grigorieva I.V. (2010). Limits on Intrinsic Magnetism in Graphene. Phys. Rev. Lett..

[26] Wang Y., Huang Y., Song Y., Zhang X., Ma Y., Liang J., Chen Y. (2009). Room-Temperature Ferromagnetism of Graphene. Nano Lett..

[27] Yazyev O.V. (2010). Emergence of Magnetism in Graphene Materials and Nanostructures. Rep. Prog. Phys..

[28] Enoki T., Takai K. (2009). The Edge State of Nanographene and the Magnetism of the Edge-State Spins. Solid State Commun..

[29] Coey J.M.D., Venkatesan M., Fitzgerald C.B., Douvalis A.P., Sanders I.S. (2002). Ferromagnetism of a graphite nodule from the Canyon Diablo meteorite. Nature.

[30] Pena Perez V., Reynaga Gonzalez C., Villegas E., Baughman J., Aguirre L., Iglesias F., Khodagulyan A., Bernal O.O., Kocharian A.N. (2025). Synthesis of Magnetic Nanostructures in Different Carbon Matrices and Post Annealing with Oxygen, Nitrogen and Argon. AIP Adv..

[31] Chilukuri B., Mazur U., Hipps K.W. (2020). Structure, Properties, and Reactivity of Porphyrins on Surfaces and Nanostructures with Periodic DFT Calculations. Appl. Sci..

[32] Heutz S., Mitra C., Wu W., Fisher A.J., Kerridge A., Stoneham M., Harker A.H., Gardener J., Tseng H.H., Jones T.S. (2007). Molecular Thin Films: A New Type of Magnetic Switch. Adv. Mater..

[33] Stephenson N.A., Bell A.T. (2007). Mechanistic Insights into Iron Porphyrin-Catalyzed Olefin Epoxidation by Hydrogen Peroxide: Factors Controlling Activity and Selectivity. J. Mol. Catal. A Chem..

[34] Li J., Jiao L., Wegener E., Richard L.L., Liu E., Zitolo A., Sougrati M.T., Mukerjee S., Zhao Z., Huang Y. (2020). Evolution Pathway from Iron Compounds to Fe1(II)–N4 Sites through Gas-Phase Iron during Pyrolysis. J. Am. Chem. Soc..

[35] Zitolo A., Goellner V., Armel V., Sougrati M.T., Mineva T., Stievano L., Fonda E., Jaouen F. (2015). Identification of Catalytic Sites for Oxygen Reduction in Iron- and Nitrogen-Doped Graphene Materials. Nat. Mater..

[36] Zhai N., Luo J., Xiao M., Zhang Y., Yan W., Xu Y. (2023). In situ construction of Co@nitrogen-doped carbon/Ni nanocomposite for broadband electromagnetic wave absorption. Carbon.

[37] Jiao Y., Dai Z., Feng M., Luo J., Xu Y. (2023). Electromagnetic absorption behavior regulation in bimetallic polyphthalocyanine-derived CoFe-alloy/C 0D/2D nanocomposites. Mater. Today Phys..

[38] Luo J., Yan W., Li X., Shu P., Mei J., Shi Y. (2023). Carbon nanotubes decorated FeNi/nitrogen-doped carbon composites for lightweight and broadband electromagnetic wave absorption. J. Mater. Sci. Technol..

[39] Mei J., Luo J., Zhao T., Jiang S., Wu Y., Dai Z., Xie Y. (2025). Morphology engineering of MIL-88A-derived 0D/1D/2D nanocomposites toward wideband microwave absorption. J. Mater. Sci. Technol..

[40] Liu W., Luo J., Shi J., Lan D., Mao S., Xie Y. (2026). Honeycomb-like BiCo@NC composites derived from bimetallic organic frameworks for high-efficiency electromagnetic wave absorption. Acta Phys.-Chim. Sin..

[41] Iglesias F., Gonzalez C.R., Baughman J., Wonderling N., Shallenberger J., Khodagulyan A., Bernal O.O., Kocharian A.N. (2024). Characterization of Carbon-Coated Core–Shell Iron Nanoparticles Annealed by Oxygen and Nitrogen. AIP Adv..

[42] Lee B., Yoon S., Lee J.W., Kim Y., Chang J., Yun J., Ro J.C., Lee J.S., Lee J.H. (2020). Statistical Characterization of the Morphologies of Nanoparticles through Machine Learning Based Electron Microscopy Image Analysis. ACS Nano.

[43] Sharma A., Kyotani T., Tomita A. (1999). A New Quantitative Approach for Microstructural Analysis of Coal Char Using HRTEM Images. Fuel.

[44] Joubert J.C., Wilke D.N., Pizette P. (2023). Fourier Image Analysis of Multiphase Interfaces to Quantify Primary Atomization. Math. Comput. Appl..

[45] Schneider C.A., Rasband W.S., Eliceiri K.W. (2012). NIH Image to ImageJ: 25 Years of Image Analysis. Nat. Methods.

[46] Schindelin J., Arganda-Carreras I., Frise E., Kaynig V., Longair M., Pietzsch T., Preibisch S., Rueden C., Saalfeld S., Schmid B. (2012). Fiji: An Open-Source Platform for Biological-Image Analysis. Nat. Methods.

[47] van der Walt S., Schönberger J.L., Nunez-Iglesias J., Boulogne F., Warner J.D., Yager N., Gouillart E., Yu T. (2014). scikit-image: Image Processing in Python. PeerJ.

[48] Potter M.E., Riley L.N., Oakley A.E., Mhembere P.M., Callison J., Raja R. (2019). The Influence of Porosity on Nanoparticle Formation in Hierarchical Aluminophosphates. Beilstein J. Nanotechnol..

[49] Zhou X., Du Y., Zhang H., Li B., Xiong X., Lv X., Yuan M., Liu Y., Chen G., Cui J. (2025). High-entropy nanoalloys anchored on entropy-compensating two-dimensional oxides for enhanced nanomagnetism. Sci. Adv..

[50] Sarkar S.K., Keskar N., Tan L.P., Ingale T., Chesetti A., Dasari S., Davidson K.P., Chaudhary V., Dahotre N., Ramanujan R.V. (2026). A magnetically soft yet mechanically strong and ductile Ta free CoFeNi high entropy alloy with Al and Ti additions. Nat. Commun..

[51] Xu X., Xiong X., Wang X., Rao L., Tan W., Yu S., Cheng H.-W., Che R. (2026). Biphasic high-entropy heterojunctions enabled by perovskite transformation. Adv. Mater..

[52] Patterson A.L. (1939). The Scherrer Formula for X-Ray Particle Size Determination. Phys. Rev..

[53] Langford J.I., Wilson A.J.C. (1978). Scherrer after Sixty Years: A Survey and Some New Results in the Determination of Crystallite Size. J. Appl. Crystallogr..

[54] Otsu N. (1979). A Threshold Selection Method from Gray-Level Histograms. IEEE Trans. Syst. Man Cybern..

[55] Sato Y., Nakajima S., Shiraga N., Atsumi H., Yoshida S., Koller T., Gerig G., Kikinis R. (1998). Three-Dimensional Multi-Scale Line Filter for Segmentation and Visualization of Curvilinear Structures in Medical Images. Med. Image Anal..

[56] Lee T.C., Kashyap R.L., Chu C.N. (1994). Building Skeleton Models via 3-D Medial Surface Axis Thinning Algorithms. CVGIP Graph. Model. Image Process..

[57] Zhang T.Y., Suen C.Y. (1984). A Fast Parallel Algorithm for Thinning Digital Patterns. Commun. ACM.

[58] Pucci C., Degl’Innocenti A., Belenli Gümüş M., Ciofani G. (2022). Superparamagnetic iron oxide nanoparticles for magnetic hyperthermia: Recent advancements, molecular effects, and future directions in the omics era. Biomater. Sci..

[59] Kacem M., Essoumhi A., Dib M. (2024). Magnetic iron oxide-based materials and their hyperthermia application: A review. Inorg. Chem. Commun..

[60] Rajan A., Laha S.S., Sahu N.K., Thorat N.D., Shankar B. (2024). Recent advancements and clinical aspects of engineered iron oxide nanoplatforms for magnetic hyperthermia-induced cancer therapy. Mater. Today Bio.

[61] Wu W., Wu Z., Yu T., Jiang C., Kim W.S. (2015). Recent Progress on Magnetic Iron Oxide Nanoparticles: Synthesis, Surface Functional Strategies and Biomedical Applications. Sci. Technol. Adv. Mater..

[62] Nguyen M.D., Tran H.V., Xu S., Lee T.R. (2021). Fe3O4 Nanoparticles: Structures, Synthesis, Magnetic Properties, Surface Functionalization, and Emerging Applications. Appl. Sci..

[63] Kaur M., McCloy J.S., Jiang W., Yao Q., Qiang Y. (2012). Size Dependence of Inter- and Intracluster Interactions in Core–Shell Iron–Iron Oxide Nanoclusters. J. Phys. Chem. C.

[64] Jun Y.w., Seo J.w., Cheon J. (2008). Nanoscaling Laws of Magnetic Nanoparticles and Their Applicabilities in Biomedical Sciences. Acc. Chem. Res..

[65] Lee J.H., Jang J.t., Choi J.S., Moon S.H., Noh S.H., Kim J.W., Kim J.G., Kim I.S., Park K.I., Cheon J. (2011). Exchange-Coupled Magnetic Nanoparticles for Efficient Heat Induction. Nat. Nanotechnol..

[66] Guardia P., Di Corato R., Lartigue L., Wilhelm C., Espinosa A., Garcia-Hernandez M., Gazeau F., Manna L., Pellegrino T. (2012). Water-Soluble Iron Oxide Nanocubes with High Values of Specific Absorption Rate for Cancer Cell Hyperthermia Treatment. ACS Nano.

[67] Abenojar E.C., Wickramasinghe S., Bas-Concepcion J., Samia A.C.S. (2016). Structural Effects on the Magnetic Hyperthermia Properties of Iron Oxide Nanoparticles. Prog. Nat. Sci. Mater. Int..

[68] Périgo E.A., Hemery G., Sandre O., Ortega D., Garaio E., Plazaola F., Teran F.J. (2015). Fundamentals and Advances in Magnetic Hyperthermia. Appl. Phys. Rev..

[69] Pankhurst Q.A., Connolly J., Jones S.K., Dobson J. (2003). Applications of Magnetic Nanoparticles in Biomedicine. J. Phys. D Appl. Phys..

[70] Pankhurst Q.A., Thanh N.T.K., Jones S.K., Dobson J. (2009). Progress in Applications of Magnetic Nanoparticles in Biomedicine. J. Phys. D Appl. Phys..

[71] Laurent S., Forge D., Port M., Roch A., Robic C., Vander Elst L., Muller R.N. (2008). Magnetic Iron Oxide Nanoparticles: Synthesis, Stabilization, Vectorization, Physicochemical Characterizations, and Biological Applications. Chem. Rev..

[72] Colombo M., Carregal-Romero S., Casula M.F., Gutiérrez L., Morales M.P., Böhm I.B., Heverhagen J.T., Prosperi D., Parak W.J. (2012). Biological Applications of Magnetic Nanoparticles. Chem. Soc. Rev..

[73] Gavilán H., Avugadda S.K., Fernández-Cabada T., Soni N., Cassani M., Mai B.T., Chantrell R., Pellegrino T. (2021). Magnetic Nanoparticles and Clusters for Magnetic Hyperthermia: Optimizing Their Heat Performance and Developing Combinatorial Therapies to Tackle Cancer. Chem. Soc. Rev..

[74] Simon P., Gogotsi Y. (2008). Materials for Electrochemical Capacitors. Nat. Mater..

[75] Sevilla M., Mokaya R. (2014). Energy Storage Applications of Activated Carbons: Supercapacitors and Hydrogen Storage. Energy Environ. Sci..

[76] Inamura I., Inamura K., Jinbo Y., Mihara T., Sasaoka Y. (2019). Preparation of Metal Phthalocyanine (MPc)–Polymer Complexes: The Possible Anti-Cancer Properties of FePc–Polymer Complexes. Heliyon.

[77] Neamtu M., Nadejde C., Brinza L., Dragos O., Gherghel D., Paul A. (2020). Iron Phthalocyanine-Sensitized Magnetic Catalysts for BPA Photodegradation. Sci. Rep..

[78] Gu X., Guo X.-B., Li W.-H., Jiang Y.-P., Liu Q.-X., Tang X.-G. (2024). High-Entropy Materials for Application: Electricity, Magnetism, and Optics. ACS Appl. Mater. Interfaces.

[79] Liu C., Yang Y., Ma Z., Zhou C., Liu D., Luo X., Zhu X., Sun Y., Sheng Z. (2020). Edge-Induced Room-Temperature Ferromagnetism in Carbon Nanosheets. J. Phys. Chem. C.

[80] Dormann J.L., Fiorani D., Tronc E. (1997). Magnetic Relaxation in Fine-Particle Systems. Advances in Chemical Physics.

[81] Tuček J., Holá K., Zoppellaro G., Błoński P., Langer R., Medved’ M., Susi T., Otyepka M., Zbořil R. (2018). Zigzag sp2 Carbon Chains Passing through an sp3 Framework: A Driving Force toward Room-Temperature Ferromagnetic Graphene. ACS Nano.

[82] Manukyan A., Gyulasaryan H., Kocharian A., Oyala P., Chumakov R., Avramenko M., Sanchez C., Bernal O.O., Bugaev L., Sharoyan E. (2022). Structure and Magnetism of Few-Layer Nanographene Clusters in Carbon Microspheres. J. Phys. Chem. C.

[83] Tuček J., Holá K., Bourlinos A.B., Błoński P., Bakandritsos A., Ugolotti J., Dubecký M., Karlický F., Ranc V., Čépe K. (2017). Room temperature organic magnets derived from sp3 functionalized graphene. Nat. Commun..

[84] Katsnelson M. (2014). Theory of Carbon-Based Magnetism. Lecture Slides. https://www.theorphys.science.ru.nl/people/katsnelson/brazil2014.pdf.

[85] Edwards D.M., Katsnelson M.I. (2006). High-temperature ferromagnetism of sp electrons in narrow impurity bands: Application to CaB6. J. Phys. Condens. Matter.

[86] Kocharyan A.N., Ovnanyan P.S. (1978). Exchange Interaction Mechanisms in Magnetic Semiconductors. Zhurnal Eksp. I Teor. Fiz..

[87] Warren B.E. (1941). X-Ray Diffraction in Random Layer Lattices. Phys. Rev..

[88] Biscoe J., Warren B.E. (1942). An X-Ray Study of Carbon Black. J. Appl. Phys..

[89] Franklin R.E. (1951). Crystallite Growth in Graphitizing and Non-Graphitizing Carbons. Proc. R. Soc. Lond. Ser. A Math. Phys. Sci..

[90] Franklin R.E. (1950). The Interpretation of Diffuse X-Ray Diagrams of Carbon. Acta Crystallogr..

